# Accurate Preoperative Distinction of Intracranial Hemangiopericytoma From Meningioma Using a Multihabitat and Multisequence-Based Radiomics Diagnostic Technique

**DOI:** 10.3389/fonc.2020.00534

**Published:** 2020-05-19

**Authors:** Jingwei Wei, Lianwang Li, Yuqi Han, Dongsheng Gu, Qian Chen, Junmei Wang, Runting Li, Jiong Zhan, Jie Tian, Dabiao Zhou

**Affiliations:** ^1^The Key Laboratory of Molecular Imaging, Chinese Academy of Sciences Institute of Automation, Beijing, China; ^2^Beijing Key Laboratory of Molecular Imaging, Beijing, China; ^3^The key Laboratory of Molecular Imaging, University of Chinese Academy of Sciences, Beijing, China; ^4^Department of Neurosurgery, Beijing Tiantan Hospital, Capital Medical University, Beijing, China; ^5^Department of Radiology, Beijing Neurosurgical Institute, Beijing, China; ^6^Department of Neuropathology, Beijing Neurosurgical Institute, Beijing, China; ^7^Advanced Innovation Center for Big Data-Based Precision Medicine, School of Medicine, Beihang University, Beijing, China; ^8^Engineering Research Center of Molecular and Neuro Imaging of Ministry of Education, School of Life Science and Technology, Xidian University, Xi'an, China; ^9^China National Clinical Research Center for Neurological Diseases, Beijing, China

**Keywords:** intracranial hemangiopericytoma, meningioma, diagnosis, magnetic resonance imaging, radiomics

## Abstract

**Background:** Intracranial hemangiopericytoma (IHPC) and meningioma are both meningeal neoplasms, but they have extremely different malignancy and outcomes. Because of their similar radiological characteristics, they are difficult to distinguish prior to surgery, leading to a high rate of misdiagnosis.

**Methods:** We enrolled 292 patients (IHPC, 155; meningiomas, 137) with complete clinic-radiological and histopathological data, from a 10-year database established at Tiantan hospital. Radiomics analysis of tumor and peritumoral edema was performed on multisequence magnetic resonance images, and a fusion radiomics signature was generated using a machine-learning strategy. By combining clinic-radiological data with the fusion radiomics signature, we developed an integrated diagnostic approach that we named the IHPC and Meningioma Diagnostic Tool (HMDT).

**Results:** The HMDT displayed remarkable diagnostic ability, with areas under the curve (AUCs) of 0.985 and 0.917 in the training and validation cohorts, respectively. The calibration curve showed excellent agreement between the diagnosis predicted by HMDT and the histological outcome, with *p*-values of 0.801 and 0.622 for the training and the validation cohorts, respectively. Cross-validation showed no statistical difference across three divisions of the cohort, with average AUCs of 0.980 and 0.941 for the training and validation cohorts, respectively. Stratification analysis showed consistent performance of the HMDT in distinguishing IHPC from highly misdiagnosed subgroups of grade I meningioma and angiomatous meningioma (AM) with AUCs of 0.913 and 0.914 in the validation cohorts for the two subgroups.

**Conclusions:** By integrating clinic-radiological information with radiomics signature, the proposed HMDT could assist in preoperative diagnosis to distinguish IHPC from meningioma, providing the basis for strategic decisions regarding surgery.

## Introduction

Intracranial hemangiopericytoma (IHPC) and meningioma are both meningeal neoplasms that share similar radiological characteristics (1). However, they have distinct histologic characteristics and biological behaviors (2–4). Unlike the majority of meningiomas, IHPC is malignant (WHO grade II–III) and has a relentless tendency to recur and metastasize (2, 4, 5). After the first relapse, sequential recurrence of IHPC is more frequent and the effectiveness of therapies decreases markedly. Hence, maximal surgical resection is imperative in the initial treatment of IHPC (6, 7). Because IHPC is highly vascularized, there is also a high risk of fatal blood loss during surgery (4).

These differences between IHPC and meningioma mean that accurate preoperative diagnosis is critically important for treatment planning. However, the high degree of overlap in the radiological characteristics has posed a great challenge for preoperative radiological diagnosis (8, 9). This challenge is also evident in the data used in this study, in which 70% of IHPCs were radiologically misdiagnosed as meningiomas and only identified by post-operative pathology analysis.

Although previous studies proved that CT/MRI-based characteristics may contribute to the diagnosis of IHPC, these are qualitative characteristics that are subject to observational bias, resulting in a high level of misdiagnosis of patients with IHPC (8, 10, 11). Imaging texture-based studies have shown that quantitative imaging features from MRI can be effective markers for distinguishing IHPCs and meningiomas (12, 13). However, studies to date lack convincing validation, and due to the small sample size, these models demonstrate only a simple correlation, which has limited clinical utility.

Radiomics, as an emerging medical image processing technique, provides a promising solution to solve this clinical problem. Radiomics can achieve the automatic extraction of high-throughput and high-dimensional imaging features from encrypted big medical imaging data (14, 15). By combining imaging information with preoperative clinical/empirical knowledge, it can identify patterns and subtypes relevant for tumor diagnosis, the evaluation of treatment effects, and prognosis (14, 16–18). Radiomics has been widely applied to predict pathological or genetic phenotypes in intra axil tumors, especially gliomas (19, 20). However, the utility of radiomics in differentiating IHPC and meningioma by multisequence MRI has yet to be established.

In this study, we conducted a retrospective analysis of high-quality data from a 10-year cohort of patients with histopathologically confirmed IHPCs and meningiomas, using multisequence and multihabitat radiomics pipeline to test the ability of radiomics to achieve high accuracy, preoperative diagnosis of IHPC and meningioma in order to assist in presurgery planning for the management and treatment of the two types of tumor.

## Materials and Methods

### Patient Enrollment

Patients were retrospectively enrolled by searching the Picture Archiving and Communications System in our hospital from January 2008 to December 2018. Clinical data were retrieved from the Electronic Medical Record. Patients were randomly split into training (*n* = 204) and validation cohorts (*n* = 88). The study was approved by the institutional review board, and all patient records and information were anonymized and de-identified. The Chinese Clinical Trial Registry identifier of the study was ChiCTR1900022671.

The inclusion criteria were as follows: (1) MR images acquired no more than 1 month before surgery; (2) preoperative standard MR imaging that included T1WI, CE-T1WI, and T2WI sequences; and (3) complete clinical records at initial diagnosis. The exclusion criteria were as follows: (1) history of craniotomy, biopsy, radiotherapy, or chemotherapy; (2) recurring tumors or multiple lesions; and (3) low-quality or unclear MRIs.

The histopathological examination and MR imaging acquisition are provided in Supplementary Appendix E1.

### Development of HMDT

Preoperative clinical and radiological information may reflect and depict different phenotypes of IHPC and meningioma; thus, we comprehensively integrated correlated clinical, radiological, and radiomics data stream into a machine learning–based model, named IHPC and Meningioma Diagnostic Tool (HMDT), to improve accurate diagnosis of IHPC and meningiomas.

#### Selection of Preoperative Clinical and Radiological Factors

A total of 14 preoperative clinical/radiological factors were analyzed as potential effective factors as reported in the references (4, 6, 8, 10, 19–24). Univariable and multivariable analyses were used to identify effective factors for the diagnosis and were integrated into a clinical model by logistic regression modeling. Detailed description of radiological factors is shown in Supplementary Appendix E2.

#### Radiomics Analysis

The radiomics analysis process was structured in three phases: radiomic feature extraction, key feature selection, and radiomics signature construction.

Initially, a set of 473 radiomic features were extracted from segmented tumor and peritumoral edema habitats using the Pyradiomics tool (https://pyradiomics.readthedocs.io). These radiomic features fall into four broad categories: shape and size, first-order statistics, textural, and wavelet features. The process of tumor segmentation is described in Supplementary Appendix E3. The detailed description of the radiomic feature definition is provided in Supplementary Appendix E4.

Feature selection was primarily conducted by assessing feature stability and reproducibility via calculating the concordance correlation coefficient (CCC) and the intraclass correlation coefficient (ICC). Multiclinician, multi-time-point, and perturbation segmentation manners for feature robustness assessment are described in Supplementary Appendix E5. We further applied the Mann–Whitney *U*-test to select diagnosis outcome-related radiomic features with a *p* < 0.05.

On the basis of this initial selection of promising variables, we then compared 64 radiomics modeling strategies including the 16 feature selection algorithms and 4 classifiers most commonly used in radiomics studies (12, 25). A detailed account of the 64 strategies is provided in Supplementary Appendix E6. Recursive feature elimination and random forest stood out as the optimal feature selection algorithm and classifier for radiomics signature construction.

The above radiomics pipeline was then conducted on T1WI-tumor, CE-T1WI-tumor, T2WI-tumor, T1WI-edema, CE-T1WI-edema, and T2WI-edema, respectively. Consequently, six radiomics signatures were acquired. A fusion radiomics signature was constructed by integrating the six single signatures by logistic regression modeling.

#### Integrated HMDT Model and Nomogram Construction

The HMDT was constructed by integrating effective clinic-radiological factors with the fusion radiomics signature. We adopted the Akaike information criterion (AIC) to select optimal incorporated factors and utilized logistic regression modeling to perform HMDT construction. In addition, a nomogram was drawn to manifest the contribution of each of the included parameters according to their weighted proportions in the model.

### Model Assessment

#### Diagnostic Performance Assessment

The diagnostic power of the proposed models was evaluated by the receiver operating characteristic (ROC) curve, area under the curve (AUC), accuracy, sensitivity, and specificity. Comparisons between AUCs were performed with the Delong test, and comparisons between specificity and sensitivity were performed by Pearson's chi-square test. Calibration curves were plotted to evaluate the calibration power of the nomogram with the Hosmer Lemeshow test. To quantify the discrimination ability of the nomogram, Harrell's C-index was calculated.

#### Assessing the Diagnostic Robustness of HMDT

To test the model robustness, we randomly divided the enrolled cohorts into training and corresponding validation cohorts three times and labeled these Groups 1, 2, and 3. The division ratio remained 7:3 for each operation. AUCs were compared using the Delong test to show whether the change of dataset would affect the performance of the HMDT. At the same time, three-fold cross-validation was performed to elude the effect of sample divisions.

#### Stratification Analysis

In light of the need to consider subpopulations in which IHPC and meningioma diagnosis is more difficult, we performed stratification analysis based on age and radiological behavior (tumor shape and dural tail sign), as well as pathological grade and subtype. Considering the majority of IHPCs that were misdiagnosed using MRI were WHO grade I meningiomas, especially angiomatous meningiomas (AMs), we conducted additional subpopulation analysis of WHO grade I meningiomas and AMs.

#### Clinical Usefulness

The clinical validity of the HMDT was assessed by decision curve analysis. Furthermore, we developed a software embedded HMDT model with a user-friendly interface. This online tool can be freely downloaded and activated using the application file provide in the reference^1^. User instructions are provided in Supplementary Appendix E7.

### Statistical Analysis

Statistical analysis was performed with PASW Statistics, version 18.0 (SPSS Inc., Chicago, IL, USA) and R software, version 3.4.1 (www.R-project.org). Statistical significance was defined with a two-sided *p* < 0.05.

## Results

### Baseline Characteristics

The baseline characteristics are summarized in Table 1. There were no significant differences between the training and validation cohorts in terms of their demographic, clinical, or radiological characteristics (*p* = 0.075–0.997).

**Table 1:** Baseline characteristics in training and validation cohorts.

**Characteristics**	**Training cohort (*****n*** **=** **204)**			**Validation cohort (*****n*** **=** **88)**			**P (inter)**
	**IHPC (*n* = 109)**	**Meningioma (*n* = 95)**	**P (intra)**	**IHPC (*n* = 46)**	**Meningioma (*n* = 42)**	**P (intra)**	
**Age** (median [IQR])	43 (31.51)	50 (39.58)	0.003	42 (28.49)	51 (38.58)	0.015	0.570
**Gender**							0.359
Male	56 (51.4)	44 (46.3)	0.471	22 (47.8)	16 (38.1)	0.357	
Female	53 (51.0)	51 (53.7)		24 (52.2)	26 (61.9)		
**Course of disease** (median [IQR])	4 (2.12)	6 (1.24)	0.063	3 (2.12)	4.5 (1.24)	0.177	0.439
**Location 1**							0.240
Frontal	44 (40.4)	64 (67.4)	<0.001	21 (45.7)	19 (45.2)	0.969	
Posterior	65 (59.6)	31 (32.6)		25 (54.3)	23 (54.8)		
**Location 2**							0.730
Supra	84 (77.1)	89 (93.7)	0.001	35 (76.1)	41 (97.6)	0.003	
Infra	25 (22.9)	6 (6.3)		11 (23.9)	1 (2.4)		
**Location 3**							0.732
Left	35 (32.1)	34 (35.8)	0.041	11 (23.9)	23 (54.8)	0.001	
Right	34 (31.2)	41 (43.2)		15 (32.6)	15 (35.7)		
Both	40 (36.7)	20 (21.1)		20 (43.5)	4 (9.5)		
**Midline type**							0.148
Yes	81 (74.3)	64 (67.4)	0.275	35 (76.1)	20 (47.6)	0.006	
No	28 (25.7)	31 (32.6)		11 (23.9)	22 (52.4)		
**Venous sinus invasion**							0.801
Yes	49 (45.0)	47 (49.5)	0.519	22 (47.8)	18 (42.9)	0.640	
No	60 (55.0)	48 (50.5)		24 (52.2)	24 (57.1)		
**Dural tail sign**							0.169
Yes	18 (16.5)	52 (54.7)	<0.001	5 (10.9)	18 (42.9)	0.001	
No	91 (83.5)	43 (45.3)		41 (89.1)	24 (57.1)		
**Tumor shape**							0.080
Regular	11 (10.1)	9 (9.5)	0.882	5 (10.9)	10 (23.8)	0.107	
Irregular	98 (89.9)	86 (90.5)		41 (89.1)	32 (76.2)		
**Enhancement pattern**							0.997
Homogeneous	25 (22.9)	19 (20.0)	0.611	10 (21.7)	9 (21.4)	0.972	
Heterogeneous	84 (77.1)	76 (80.0)		36 (78.3)	33 (78.6)		
**Tumor margin**							0.314
Clear	20 (18.3)	31 (32.6)	0.019	11 (23.9)	16 (38.1)	0.150	
Unclear	89 (81.7)	64 (67.4)		35 (76.1)	26 (61.9)		
**Peritumoral edema**							0.075
Absent	25 (22.9)	13 (13.7)	0.007	16 (34.8)	11 (26.2)	0.052	
Moderate	60 (63.2)	75 (68.8)		28 (60.9)	22 (52.4)		
Extensive	9 (8.3)	22 (23.2)		2 (4.3)	9 (21.4)		
**Serpentine signal voids**							0.699
Yes	96 (88.1)	68 (71.6)	0.003	40 (87.0)	29 (69.0)	0.041	
No	13 (11.9)	27 (28.4)		6 (13.0)	13 (31.0)		

*IHPC, intracranial hemangiopericytoma; P (Intra) is the result of univariable analyses between methylated and unmethylated groups; P (Inter) represents whether there exists significant difference between training and validation cohorts; IQR represents the interquartile range. Unless otherwise specified, data are numbers of patients, with percentages in parentheses*.

**Figure 1:**
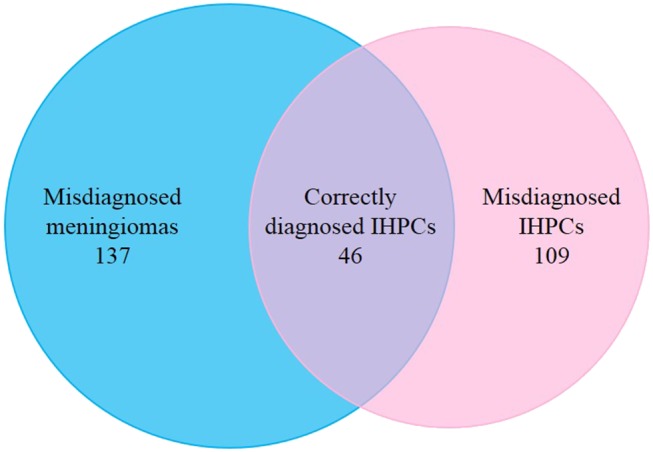
Distribution of enrolled patients. Left circle represents cases of radiologically diagnosed IHPCs; right circle represents cases of pathologically diagnosed IHPCs. Intersection (purple) of two circles represents 46 cases of enrolled IHPCs, which were correctly diagnosed by radiology; 137 cases of pathologically diagnosed meningiomas were radiologically misdiagnosed as IHPCs (blue); 109 cases of pathological diagnosed IHPCs were radiologically misdiagnosed as meningiomas (pink).

A total of 292 cases were enrolled in this study, of which 137 cases were pathologically diagnosed as meningiomas and 155 cases were pathologically diagnosed as IHPCs. Radiologically, all the enrolled meningiomas were misdiagnosed as IHPCs and 109 enrolled IHPCs were misdiagnosed as meningiomas. Only the remaining 46 cases of enrolled IHPCs were correctly radiologically diagnosed (Figure 1). Based on the 2016 WHO classification of CNS tumors, the pathological grades of these patients were as follows: 97 WHO grade II IHPCs, 58 WHO grade III IHPCs, 112 WHO grade I meningiomas, 22 WHO grade II meningiomas, and 3 WHO grade III meningiomas. There was no significant difference in the distribution of IHPC and meningioma between the training and validation cohorts (*p* = 0.856).

### Selected Clinic-Radiological Factors

Seven clinical/radiological factors were selected as effective diagnostic parameters, which were the course of disease, location (frontal/posterior), location (supra/infra), dural tail sign, tumor margin, peritumoral edema, and serpentine signal voids. The AUC for each single clinic-radiological factor turned out to be <0.6 in the validation cohort. The result of uni- and multivariable analysis and AUC of each selected factor is shown in Supplementary Table 1.

### Diagnostic Performance of the Clinical Model

**Figure 2:**
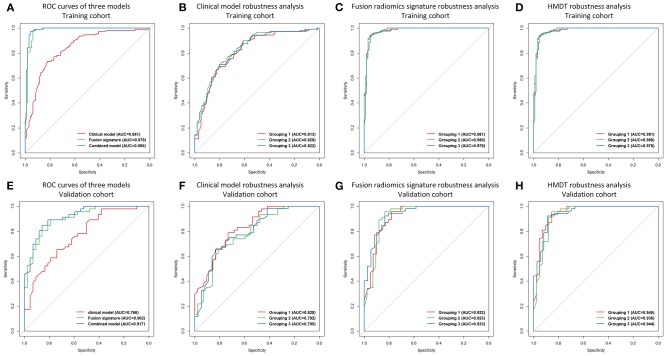
ROC curves and robustness analysis results. ROC curves of the clinical model, the fusion radiomics signature, and the HMDT in the training cohort were shown in **(A)**, and the ROC curves of the three models in the validation cohort were shown in **(E)**. Robustness analysis for the clinical model, the fusion radiomics signature, and the HMDT in the training cohort were shown in **(B–D)**, respectively. For the validation cohort, robustness analysis for the three models were shown in **(F–H)**, respectively.

Combining the seven single clinical/radiological factors into a multiparametric clinical model significantly increased the diagnostic power (training: *p* < 0.001; validation: *p* = 0.002). The AUCs of the clinical model were 0.841 and 0.766 in the training and validation cohorts, respectively. Detailed predictive indicators, the ROC curve, and the violin graph of the clinical model are shown in Table 2, Figure 2, and Supplementary Figure 1, respectively.

**Table 2:** Diagnostic ability of the developed models.

**Model**	**Training cohort (*****n*** **=** **204)**				**Validation cohort (*****n*** **=** **88)**			
	**AUC (95% CI)**	**ACC**	**SEN**	**SPE**	**AUC (95% CI)**	**ACC**	**SEN**	**SPE**
Clinical model	0.841 (0.787, 0.896)	0.760	0.734	0.790	0.766 (0.667, 0.863)	0.659	0.674	0.643
T1_tumor signature	0.859 (0.810, 0.908)	0.819	0.972	0.642	0.818 (0.732, 0.904)	0.716	0.891	0.524
T1_edema signature	0.768 (0.706, 0.829)	0.716	0.927	0.474	0.673 (0.569, 0.777)	0.648	0.957	0.310
T2_tumor signature	0.858 (0.809, 0.907)	0.794	0.927	0.642	0.762 (0.666, 0.858)	0.693	0.849	0.524
T2_edema signature	0.787 (0.727, 0.846)	0.750	0.936	0.537	0.711 (0.613, 0.809)	0.682	0.957	0.381
CE-T1_tumor signature	0.811 (0.752, 0.870)	0.755	0.725	0.789	0.731 (0.628, 0.835)	0.648	0.630	0.667
CE-T1_edema signature	0.760 (0.699, 0.821)	0.770	0.982	0.526	0.734 (0.652, 0.817)	0.659	1.000	0.286
Tumor signature	0.917 (0.877,0.958)	0.878	0.973	0.768	0.872 (0.799, 0.944)	0.750	0.848	0.643
Edema signature	0.808 (0.747, 0.869)	0.775	0.973	0.547	0.704 (0.597, 0.811)	0.659	0.978	0.310
Fusion signature	0.979 (0.959, 0.999)	0.956	0.991	0.916	0.902 (0.841, 0.964)	0.818	0.891	0.738
HMDT	0.985 (0.968, 1)	0.961	0.973	0.947	0.917 (0.861, 0.972)	0.852	0.848	0.857

*AUC, area under the curve; CI, confidence interval; ACC, accuracy; SEN, sensitivity; SPE, specificity*.

### Diagnostic Performance of Quantitative Radiomics Signatures

When combining the six single radiomics signatures, the fusion signature reached satisfactory AUCs of 0.979 and 0.902 in the training and validation cohorts, respectively. The process of feature selection is shown in Supplementary Table 2. The selected radiomic features and their diagnostic performance are shown in Supplementary Table 3. Detailed predictive indicators, the ROC curve, and the violin graph of the radiomics signatures are shown in Table 2, Figure 2, and Supplementary Figure 1, respectively. The results from the 64 modeling strategies are shown in Supplementary Table 4. Decision trees of the six single radiomics signatures are shown in Supplementary Figure 2.

### Diagnostic Performance of HMDT

The final integrated HMDT model produced extremely accurate diagnosis of IHPC and meningioma with AUCs of 0.985 and 0.917 in the training and validation cohorts, respectively. The HMDT showed a significant improvement in diagnostic power over the clinical model, with *p* < 0.001 and 0.002 in the training and validation cohorts, respectively. Comparing of the HMDT and the fusion radiomics signature showed a numerical increase, but it was not statistically significant in either the training (*p* = 0.141) or validation (*p* = 0.133) cohorts. Detailed predictive indicators, the ROC curve, and the violin graph of the HMDT are shown Table 2, Figure 2, and Supplementary Figure 1, respectively. The heatmap showing the correlation between clinical factors and the selected radiomic features is shown in Supplementary Figure 3.

### Robustness of HMDT

In the three randomly assigned training and validation subcohorts, the ROC curves for the clinical model, fusion radiomics signature, and the HMDT overlapped (Figure 2). The Delong test showed that there were no significant differences among the three subcohorts with a *p*-value all larger than 0.05. It revealed the robustness of the modeling process and the consistent performance of the models regardless of changes in the cohorts. Detailed performance indicators of the three models are shown in Supplementary Table 5. The result of the three-fold cross-validation is shown in Supplementary Table 6.

### Stratification in Difficult-to-Diagnosis Subpopulations

In stratification analysis, the HMDT presented with satisfactory diagnostic power across subpopulations (Table 3). Importantly, in the highly misdiagnosed WHO grade I meningioma group, the HMDT still showed superior diagnostic ability with a high AUC of 0.988/0.914. Surprisingly, the HMDT demonstrated equally satisfactory diagnostic power even in the especially hard to diagnose AM group, with an AUC of 0.997/0.913.

**Table 3:** Stratification analysis of HMDT on training and validation cohorts.

**Subpopulation**	**Training cohort (*****n*** **=** **204)**				**Validation cohort (*****n*** **=** **88)**			
	**AUC (95% CI)**	**ACC**	**SEN**	**SPE**	**AUC (95% CI)**	**ACC**	**SEN**	**SPE**
**Age**								
<44	0.971 (0.921, 1)	0.941	0.946	0.933	0.894 (0.797, 0.990)	0.829	0.846	0.800
≥44	0.991 (0.948, 1)	0.966	0.982	0.954	0.933 (0.865, 1)	0.894	0.900	0.889
**Tumor shape**								
Yes	1	0.950	1	0.889	0.930 (0.805, 1)	0.667	1	0.500
No	0.983 (0.963,1)	0.962	0.980	0.941	0.924 (0.866,0.982)	0.863	0.829	0.906
**Dural tail sign**								
Yes	0.983 (0.957, 1)	0.929	1	0.904	0.944 (0.849, 1)	0.739	1	0.667
No	0.978 (0.945, 1)	0.970	0.989	0.930	0.904 (0.931, 0.976)	0.831	0.878	0.750
**WHO grade I meningiomas**	0.988 (0.968, 1)	0.968	0.973	0.961	0.914 (0.854, 0.973)	0.854	0.848	0.861
**Angiomatous meningiomas**	0.997 (0.992, 1)	0.978	0.991	0.920	0.913 (0.816, 1)	0.873	0.935	0.556

*AUC, area under the curve; CI, confidence interval; ACC, accuracy; SEN, sensitivity; SPE, specificity*.

### Nomogram and Software Development for Clinical Use

**Figure 3:**
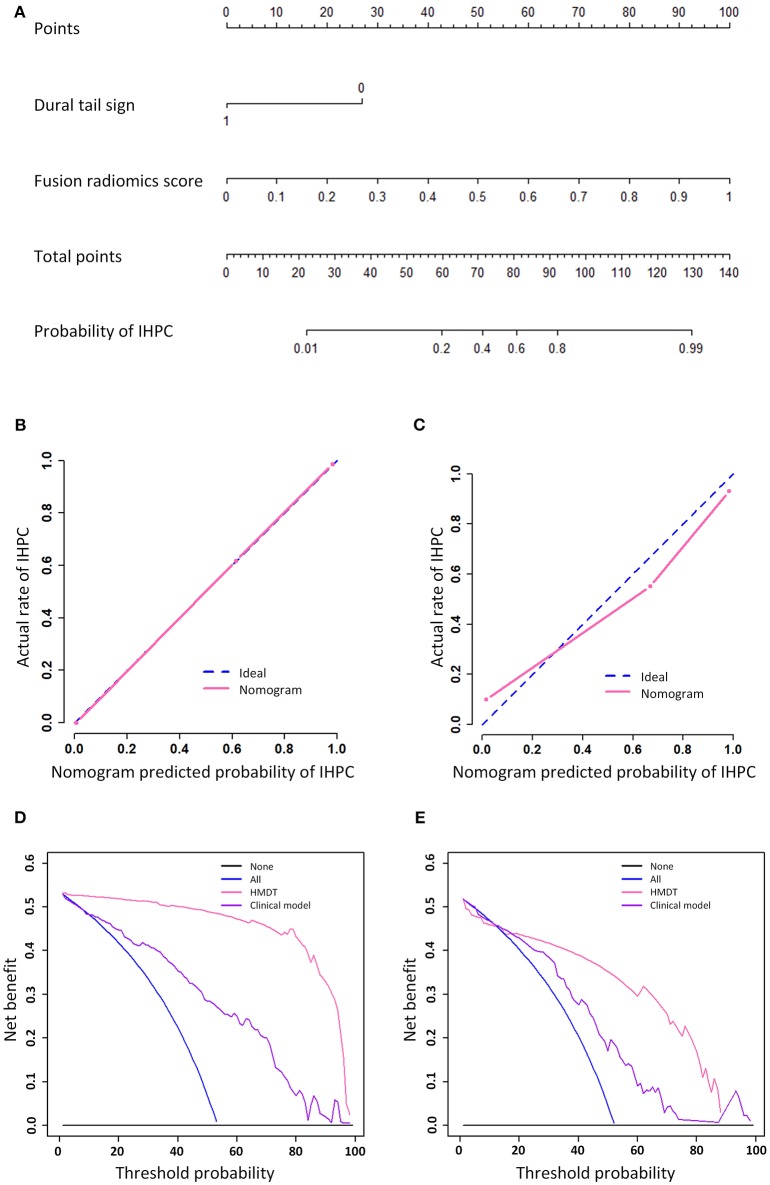
Nomogram, calibration curve, and decision analysis curve. The nomogram that showed a linear presentation of the HMDT was shown in **(A)**. The calibration curves in training and validation cohorts were shown in **(B,C)**, respectively. Decision analysis curves in the training and validation cohorts were shown in **(D,E)**. The y-axis represents the net benefit and the x-axis represents the threshold probability.

The graphical nomogram is shown in Figure 3. The Hosmer Lemeshow test yielded no significant difference between the outcomes predicted by the HMDT and the actual histopathological outcomes with a *p* < 0.801 and 0.622 in the training and validation cohorts, respectively. Decision curve analysis showed that the HMDT performed with a net improvement of 0.21% with cutoff probability of 0% in the training cohort and 0.19% improvement with 18% cutoff probability in the validation cohort. Furthermore, examples for cases diagnosed using the developed HMDT online tool are provided in Supplementary Appendix E7.

### Typical Case Analysis

**Figure 4:**
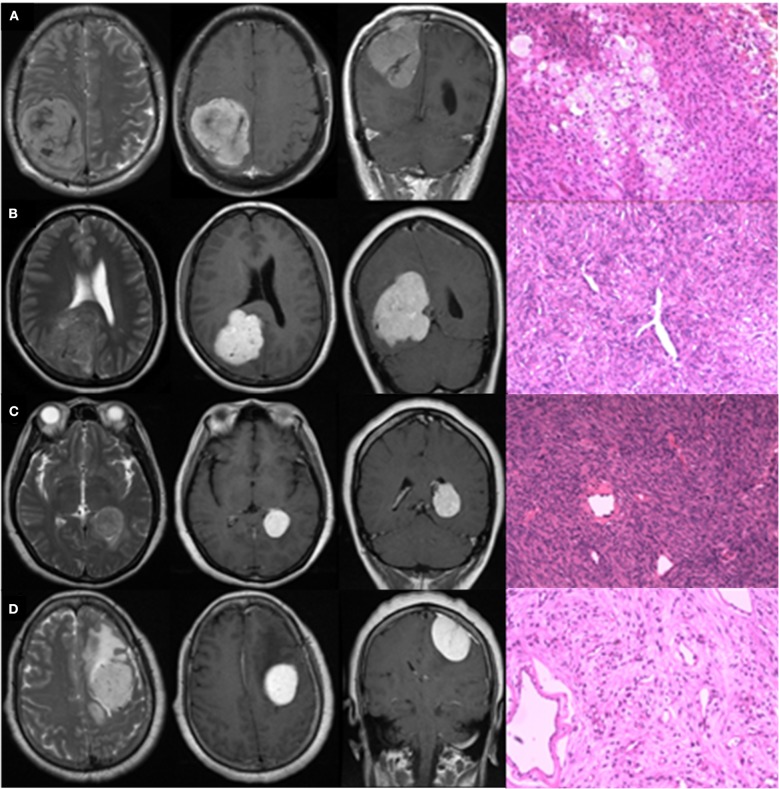
Typical radiologically misdiagnosed cases. The four graphs in row were T2WI in axial view, CE-T1WI in axial and coronal view, and pathological image, respectively. Lesions in Cases **(A,B)** were supratentorial, posterior, and close to midline in location with internal serpentine signal voids, and absence of peritumoral edema on T2WI; irregular shape, unclear margin, and absence of the dural tail sign on CE-T1WI. Enhancement in Case **(A)** was heterogeneous, while in Case **(B)**, it was homogeneous. Lesions in Cases **(C,D)** were supratentorial and lateral in location with extensive peritumoral edema on T2WI; homogeneous enhancement, regular shape, and clear margin on CE-T1WI. Lesion in Case **(C)** grew in the lateral ventricle without apparent blood supply. Lesion in Case **(D)** located in the frontal, presenting with internal serpentine signal voids and clear dural tail sign. Actually, Cases **(A,D)** were pathologically confirmed meningiomas; Cases **(B,C)** were pathologically confirmed IHPCs.

Figure 4 presents four typical cases and description of their radiological characteristics. Cases A and B were strongly suspected to be IHPCs on the basis of radiological information, but pathological analysis later found that Case A was meningioma. Cases C and D were strongly suspected to be meningiomas, but Case C was later found to be IHPC. The HMDT successfully diagnosed the four cases in accordance with the pathological results, with high probabilities. The probabilities that Cases A and D would be IHPCs were 7.8 and 0.1%, respectively, and the probabilities that Cases B and C would be IHPCs were 98.2 and 98.4%, respectively. Other results predicted by the HMDT are shown in Supplementary Table 7.

As a further blind test of the added value of the HMDT over diagnoses based on current clinical practice, we asked five junior neurosurgeons (working experience <5 years), two senior neurosurgeons (working experience>10 years), and one expert (working experience>30 years) to distinguish IHPC and meningioma for the four typical cases above. As expected, seven out of eight neurosurgeons wrongly diagnosed Case A as IHPC, and none of the eight neurosurgeons correctly diagnosed Case C as IHPC. The expert double-wrongly diagnosed Cases A and C. The diagnosed results of the eight neurosurgeons for the four cases are shown in Supplementary Table 8.

## Discussion

In this retrospective study, we explored the power of multihabitat and multisequence based radiomics for IHPC and meningioma preoperative diagnosis. The proposed effective tool, the HMDT, was developed by integrating clinic-radiological factors and the fusion radiomics signature. The HMDT improved the diagnostic accuracy with a high AUC of 0.985 in the training cohort and 0.917 in the validation cohort, which could enable a more reliable pretherapy diagnostic basis for subsequent treatment strategy making.

Over the past 30 years, previous studies exploring the use of radiological and/or clinical information in IHPC and meningioma preoperative diagnosis have shown some progress. He et al. have proved that the apparent diffusion coefficient (ADC) value was efficient for IHPC and AM (26). However, they concluded that conventional MRI and clinical factors fail to correlate with the pathological classification of IHPC and AM. Our rigorously proposed fusion radiomics signature derived from conventional MRI achieved superior performance with an AUC of 0.913 for IHPC and AM diagnosis, which was a significant improvement over the diagnostic power of the ADC value, which had an AUC of only 0.86. We acknowledge that, as other studies have shown, the ADC value contributes to IHPC and meningioma diagnosis (21, 26). However, in this paper, we demonstrate the unexploited power of conventional MRI data. Both conventional and functional MRI data should be fully utilized to increase the radiological diagnosis accuracy for IHPC and meningioma.

In our study, the dural tail sign was incorporated as the only clinic-radiological factor in the HMDT model. Previous studies have proved that narrow-based dural attachment and the absence of a dural tail sign were distinguishable factors in the diagnosis of IHPC and meningioma (8, 10, 11, 27). In our study, the dural tail sign presented a statistically different distribution in IHPCs and meningiomas with a *p* < 0.01. This was in concordance with previous results. Because the dural tail sign was associated with the chronic stimulation of meninges by the dural-attached lesion, although IHPC and meningioma are both dural-based tumors, their entirely different origin, growth rate, and malignancy may lead to such diversified manifestations of dural attachment and dural tail sign (27). Integrating the dural tail sign into the HMDT did not significantly improve its diagnostic ability, which implies that the quantified fusion radiomics signature has a more significant role in diagnosis than previously reported qualitative radiological factors. However, although it did not show a significant increase, adding the dural tail sign into the HMDT did boost its numerical accuracy. This shows the advantage of the integrated HMDT over the conventional radiological factor and single fusion radiomics signatures.

With regard to radiomic features, our results showed that the majority of selected features turned out to be wavelet features, which reflected multiscale information relating to tumor/edema areas. Through scale and translation operations, wavelet transformation could provide details focused on either high-frequency or low-frequency domains, leading it to be termed a “microscope in mathematics.” Interestingly, we found that for tumor habitat on CE-T1WI, 9 out of 20 features were two-dimensional high-frequency transformation-based features. These features described the edge and details of the tumor region. Consistent with existing knowledge, after injection of Gd-DTPA, CE-T1WI could clearly display detailed radiological edge and intratumoral information including the boundary between the tumor and the normal brain tissue, intratumoral micronecrosis, blood supply, and capillary permeability. In terms of the tumor habitat on T2WI, 8 out of 20 features were, on the contrary, two-dimensional low-frequency transformation-based features. These features provided a general view of the tumor, but did not capture its detailed characteristics. Not surprisingly, compared with CE-T1WI, T2WI focused more on the peritumoral edema area. Because of the lower contrast between the edema area and surrounding lesions/tissues, edge information would be weaker on T2WI, but it otherwise displayed the general intensity distribution of the ROI.

In our sample cohort, the majority of cases misdiagnosed as IHPCs were WHO grade I meningiomas, and AMs account for one third of these meningiomas. Thus, WHO grade I meningiomas, especially AMs, should be further stratified as a distinct subgroup. Previous studies have also pointed out the difficulty of AM and IHPC diagnosis (28–30). In this respect, the HMDT exceeded our expectations, displaying extremely satisfactory diagnostic ability with an AUC of 0.914 and 0.913 in the validation cohorts for WHO grade I meningioma and AM diagnosis, respectively. This showed that the HMDT not only successfully distinguishes IHPC and meningioma in the overall population but also can accurately diagnose IHPC in difficult cases, providing excellent preoperative guidance for clinicians.

Although this study achieved exciting initial results, a couple of limitations should be mentioned. First, only conventional MR sequences were used in the analysis. Functional MRI data are worthy of further exploration. Second, with a larger sample size, deep learning-based radiomics could be further applied. Third, manual segmentation to draw the tumor lesion was time consuming and labor intensive. Semiautomatic segmentation algorithms should be explored via a neuronetwork on both tumor and peritumoral edema areas.

In conclusion, the HMDT developed as the result of this study can realize high-accuracy diagnosis for IHPC and meningioma through machine learning–based radiomics analysis. The study results indicate that there is no doubt that the HMDT can be used as a clinical tool that has excellent robustness and subpopulation diagnostic power, and that will significantly improve the preoperative diagnosis of IHPC and meningioma, providing crucial information for the planning of subsequent treatment.

## Data Availability Statement

All datasets generated for this study are included in the article/Supplementary Material.

## Ethics Statement

The studies involving human participants were reviewed and approved by Ethics Committee of the Beijing Tiantan Hospital, Capital Medical University, Beijing, China. Written informed consent for participation was not required for this study in accordance with the national legislation and the institutional requirements.

## Author Contributions

JWe and YH implemented the algorithm and software development, literature searching, and manuscript writing. LL implemented data collecting, ROI segmentation, literature searching, and manuscript writing. DG contributed to data analysis and figures making. QC and JZ identified the radiological characteristics of IHPCs and meningiomas, and estimated and adjusted the accuracy of ROIs. JWa reviewed and confirmed the pathological diagnosis of IHPC and meningiomas according to the 2016 WHO classification of CNS tumors. RL contributed to data collecting and ROI segmentation. DZ and JT conducted the design, quality control, and data interpretation of this study.

## Conflict of Interest

The authors declare that the research was conducted in the absence of any commercial or financial relationships that could be construed as a potential conflict of interest.

## Footnotes

^1^Available online at: http://www.radiomics.net.cn/post/118 (2019).
