# Supplementary material for: Accurate Preoperative Distinction of Intracranial Hemangiopericytoma From Meningioma Using a Multihabitat and Multisequence-Based Radiomics Diagnostic Technique
Source: Front Oncol. 2020 May 19;10:534. doi: 10.3389/fonc.2020.00534 (PMC7248296; doi:10.3389/fonc.2020.00534)
Supplement: Supplementary file 1 [file Data_Sheet_1.docx]

**Supplementary Appendix**

**E1: The histopathological examination, and MR imaging acquisition**

**Histopathological examination**

All specimens obtained from surgery were fixed in 4% neutral formalin and embedded into paraffin. Each sample was cut into 5$\mu$m thickness slices and routinely stained by hematoxylin and eosin for light microscopy. The definite diagnosis of histopathology of IHPC and meningioma were confirmed by an expert histopathologists (JMW) with over 20 years working experience based on the 2016 WHO classification of CNS tumors. The main feature of IHPC was the presence of numerous slit-like capillaries, flat endothelia lining, and well-developed reticular sheath. Meningiomas exhibit various histological behaviors because of their complex pathological subtypes, including meningothelial, fibrous, transitional meningiomas, etc. The majority were diagnosed with the constituent of neoplastic arachnoidal (meningothelial) cells or spindle cells.

**MR imaging**

Preoperative MRI including sequences T1WI, CE-T1WI, and T2WI were analyzed in this study. MR images were performed using a 1.5T (n=42, Signa HDe, GE Medical System), or a 3.0T (n=250, Magnetom Verio or Trio Tim, Siemens Healthcare, Erlangen, Germany) device and the slice interval gaps were all set to 5 millimeters. In-plane resolution was ≤1 mm. The acquisition parameters including Echo times (TE), repetition times (TR), and field of view (FOV) were as follows: axial T1 weighted (10ms, 2400ms, 512×512mm at 1.5T; 10ms, 2000ms, 512×432mm at 3.0T), axial contrast-enhanced T1 weighted image (10ms, 2400ms, 512×512mm at 1.5T; 10ms, 2000ms, 512×432mm at 3.0T) after gadopentate dimeglumine (Gd-DTPA Beijing Beilu Pharmaceutical Co., Beijing China) injection; axial T2 weighted (117ms, 6000ms, 512×512mm at 1.5T; 84ms, 4500ms, 384×324mm at 3.0T). The parameters of these lesions slightly differed according to filed strength and clinical demands. To reduce the confounding variability of imaging acquisition, we utilized z-score standardization to normalize images where image intensity was scaled to 0-1.

**E2: Radiological characteristics**

The qualitative radiological features contained location, venous sinus invasion, dural tail sign, tumor shape, enhancement pattern, tumor margin, peritumoral edema and serpentine signal voids. The cranial cavity was divided into frontal/posterior, left/right/both, supra/infra sides according to the Taylor-Haughton line (from nasion to external occipital protuberance), posterior ear line (perpendicular to the baseline through the mastoid process), and tentorium, respectively. Tumors growing near the Taylor-Haughton line were regarded as midline type, otherwise as lateral type. For dural tail sign, the maximum diameter of dural attachment on CE-T1WI was larger than that of tumor and displays as linear enhancement like a rat’ tail, which was defined as dural tail sign. Tumor shape was defined as regular if round or oval shape, irregular if mushroom or lobulated shape. After injection of Gd-DTPA, enhancement pattern was defined as heterogeneous if internal necrosis existed, otherwise homogeneous. The lesion with sharp boundary between surrounding tissues was considered as clear margin, while blurred boundary or invasion of surrounding tissues as unclear margin. Peritumoral edema was categorized into absent, moderate (maximum width < maximum tumor diameter) and extensive (maximum width > maximum tumor diameter). The serpentine signal voids were defined as internal hypo-intensity signal on T2WI, which suggested large vessels. To make the process as objective as possible, the characteristics were firstly determined by two neurosurgeons (LWL and RTL), respectively, who were blinded to the radiological and pathological diagnoses. For cases with different opinions, the features were re-determined by an experienced radiologist and reviewed by a radiological expert as the final judgement. The two radiologists were also blinded to the radiological and pathological diagnoses.

**E3: Manual Tumor Segmentation**

The NIfTI data of T1WI and T2WI was registered to CE-T1WI using a mutual information based algorithm with a 12-degrees-of-freedom transformation in the FSL Linear Image Registration Tool (FSL-FLIRT) (FMRIB, Oxford, UK: <http://www.fmrib.ox.ac.uk/fsl/>) to accomplish spatial normalization. Manual segmentation was performed by a public software ITK-SNAP (<http://www.radiantviewer.com>) in a blinded fashion by 2 board-certified neurosurgeons (LWL and RTL) with 5 years of work experience, respectively. Due to the apparent enhancement on CE-T1WI and clear presentation of peritumoral edema on T2WI, regions of interest (ROIs) were delineated on tumor based on CE-T1WI, and delineated on peritumoral edema based on T2WI. Automatic registration by FSL-FLIRT was unsuccessful in 49 cases, manual segmentation of tumor and peritumoral edema were performed on T1WI, CE-T1WI, and T2WI, respectively. Meanwhile, the segmented lesion should meet the following criteria: 1) lesions should be measured with diameter larger than 1cm in at least one dimension by MRI; 2) ROI of tumor and peritumoral edema delineated on CE-T1WI and T2WI in registered cases should be in consistent with corresponding regions in other sequences. Final ROI was determined as the overlapped area of the segmentation results from the two radiologists, and each ROI was reviewed by 2 senior neuroradiologist (JZ and QC) with 20 years of working experience.

**E4: Radiomic feature definitions**

In this study, we totally extracted 473 radiomic features that could be divided into 4 types: shape and size, first-order statistics, textural, and wavelet features. Shape and size features (n=13) described three-dimensional shape and size of the tumor lesion. First order statistics features (n=18) described the histogram distribution of the voxel intensities contained within the ROI. Textural features described the heterogeneous texture patterns of the tumor lesion based on Gray Level Co-Occurrence (GLCM, n=23), Gray Level Run-Length (GLRLM, n=16), Gray Level Size-Zone (GLSZM, n=16), Gray Level Distance-Zone (GLDZM, n=14), and Neighborhood Gray Tone Difference (NGTDM, n=5) texture matrixes.

Besides the original CT image, 4 filtered images were acquired by decomposing the original image in low- and high-frequencies using a two-dimensional wavelet transformation. Shape and size features were extracted on original images. First order and textural features were extracted on both original and wavelet filtered images.

Detailed definition and formula of the features were listed as follows:

(A) Shape and size features (n=16)

Geometric features make description of the shape and size characteristics of the tumor. Let V denote the volume and A the surface area of the volume of interest (VOI). Let N denote the total number of voxels, X = {$\vec{X_{1}}, \vec{X_{2}}, \ldots,\vec{X_{N}}$} the set of Cartesian coordinate vectors and I = {I_1_, I_2_, …, I_N_}the corresponding intensity values. Detailed geometric feature formulas are as follows:

(1) Maximum diameter

The maximum diameter is defined as the largest pairwise Euclidean distance on the surface of the VOI [1]. It includes four types of maximum diameter:

The maximum three-dimensional tumor diameter

The maximum two-dimensional diameter on all transversal planes

The maximum two-dimensional diameter on all sagittal planes

The maximum two-dimensional diameter on all coronal planes

(2) Major axis length

The axis lengths are defined as extends of the volume along the three principle axis [1]. Principle component analysis on the x, y and z coordinates of all voxels on the border is used to determine the three orthogonal eigenvectors and corresponding eigenvalues (λ_max_, λ_minor_, λ_min_). The major axis length is the largest eigenvalue λ_max_.

(3) Minor axis length

The second largest eigenvalue λ_major_.

(4) Least axis length

The smallest eigenvalue λ_min_.

(5) Elongation

$$elongation=\frac{\lambda_{\mathrm{minor}}}{\lambda_{\max}}$$

(6) Flatness

$$flatness=\frac{\lambda_{\min}}{\lambda_{\max}}$$

(7) Sphericity

$$sphericity=\frac{\pi^{\frac{1}{3}}\left( 6V \right)^{\frac{2}{3}}}{A}$$

(8) Surface area

The surface area of the tumor is defined as the number of voxels on the border of the tumor.

(9) Volume

The volume of the tumor is defined as the number of voxels within the volume.

(10) Surface to volume ratio

$$surface to volume ratio=\frac{A}{V}$$

(B) First order statistics features (n=18)

First order statistics features make description of the intensity distribution of voxels in the VOI. Let **X** denote the image matrix with N voxels, **P** the first order histogram with N_l_ discrete intensity bins. Detailed first order statistics feature formulas are as follows:

(1) Energy

$$energy=\sum_{i=1}^{N} {X(i)}^{2}$$

(2) Entropy

$$entropy=\sum_{i=1}^{N_{l}} P\left( i \right)\log_{2}P(i)$$

(3) Kurtosis

$$kurtosis=\frac{\frac{1}{N}\sum_{i=1}^{N} {(X\left( i \right)-\bar{X})}^{4}}{\left( \frac{1}{N}\sum_{i=1}^{N} {(X\left( i \right)-\bar{X})}^{2} \right)^{2}}$$

(4) Maximum

$$maximum=max\mathbf{(X})$$

(5) Mean

$$mean=\bar{X}=\frac{1}{N}\sum_{i=1}^{N} X(i)$$

(6) Mean absolute deviation

$$mean absolute deviation=\frac{1}{N}\sum_{i=1}^{N} |X\left( i \right)-\bar{X}|$$

(7) Median

The sample median of **X**, or the 50th percentile of **X**.

(8) Minimum

$$minimum=min(\mathbf{X})$$

(9) Range

$$range=\max\left( \mathbf{X} \right)-min(\mathbf{X})$$

(10) Root mean square (RMS)

$$RMS=\sqrt{\frac{\sum_{i}^{N} {X(i)}^{2}}{N}}$$

(11) Skewness

$$skewness=\frac{\frac{1}{N}\sum_{i=1}^{N} {(X\left( i \right)-\bar{X})}^{3}}{\left( \sqrt{\frac{1}{N}\sum_{i=1}^{N} {(X\left( i \right)-\bar{X})}^{2}} \right)^{3}}$$

(12) Standard deviation

(13) Robust mean absolute deviation

The mean absolute of those voxels in X with a gray-level between the 10^th^ and 90^th^ percentile.

(14) 10^th^ percentile

The 10^th^ percentile of X, a robust alternative to the minimum gray-value.

(15) 90^th^ percentile

The 90^th^ percentile of X, a robust alternative to the maximum gray-value.

(16) Interquartile range

The interquartile range is defined as the 75^th^ minus the 25^th^ percentile of X.

(17) Uniformity

$$uniformity=\sum_{i=1}^{N_{l}} {P(i)}^{2}$$

(18) Variance

$$variance=\frac{1}{N-1}\sum_{i=1}^{N} {(X\left( i \right)-\bar{X})}^{2}$$

(C) Textural features (74)

Textural features make description of intrinsic heterogeneous texture of the tumor lesion. A total of 5 texture matrix were constructed after discretizing the voxel intensity within VOI by resampling the voxel intensities into equal spaced bins with a bin-width of 25 Hounsfield Units (HUs). The features extracted based on corresponding texture matrix are as follows:

1) GLCM textural features (n=23)

GLCM makes description of the second joint probability function of an image. P(i, j; θ, α) denotes the GLCM, where the (i, j)^th^ element represents two pixels with intensity levels i and j occurring at a distance of θ=1 in 13 directions.

N_g_ is the number of discrete intensity levels in the image,

μ is the mean of P(i, j),

$p_{x}\left( i \right)=\sum_{j=1}^{N_{g}} P(i,j)$depicts the marginal row probabilities,

$p_{y}\left( j \right)=\sum_{i=1}^{N_{g}} P(i,j)$ depicts the marginal column probabilities,

$\mu_{x}$(i) is the mean of $P_{x}$(i), $\mu_{x}=\sum_{i=1}^{N_{g}} ip_{x}\left( i \right)$

$\mu_{y}$(i) is the mean of $P_{y}$(i), $\mu_{y}=\sum_{j=1}^{N_{g}} jp_{y}\left( j \right)$

$\sigma_{x}$ is the standard deviation of p_x_,

$\sigma_{y}$is the standard deviation of p_y_,

$p_{x+y}\left( k \right)=\sum_{i=1}^{N_{g}} \sum_{j=1}^{N_{g}} P\left( i,j \right)$, i+j=k,k=2,3,…,2 N_g_,

$p_{x-y}\left( k \right)=\sum_{i=1}^{N_{g}} \sum_{j=1}^{N_{g}} P\left( i,j \right)$, |i-j|=k, k=0,1,…,N_g_-1,

$HX=-\sum_{i=1}^{N_{g}} p_{x}\left( i \right)\log_{2} \left[ p_{x}\left( i \right) \right]$ is the entropy of p_x_,

$HY=-\sum_{i=1}^{N_{g}} p_{y}(i)\log_{2} \left[ p_{y}(i) \right]$ is the entropy of p_y_,

$H=-\sum_{i=1}^{N_{g}} \sum_{j=1}^{N_{g}} P(i,j)\log_{2} \left[ P(i,j) \right]$is the entropy of P(i,j),

$HXY1=-\sum_{i=1}^{N_{g}} \sum_{j=1}^{N_{g}} P(i,j)\log_{2} (p_{x}(i)p_{y}(j))$,

HXY2 = $-\sum_{i=1}^{N_{g}} \sum_{j=1}^{N_{g}} p_{x}\left( i \right)p_{y}(i)\log_{2} (p_{x}(i)p_{y}(j))$,

(1) Autocorrelation

$$autocorrelation=\sum_{i=1}^{N_{g}} \sum_{j=1}^{N_{g}} ijP(i,j)$$

(2) Cluster prominence

$$cluster prominence=\sum_{i=1}^{N_{g}} \sum_{j=1}^{N_{g}} \left[ i+j-\mu_{x} \left( i \right)-\mu_{y} \left( j \right) \right]^{4}P(i,j)$$

(3) Cluster shade

$$cluster shade=\sum_{i=1}^{N_{g}} \sum_{j=1}^{N_{g}} \left[ i+j-\mu_{x} \left( i \right)-\mu_{y} \left( j \right) \right]^{3}P(i,j)$$

(4) Cluster tendency

$$cluster tendency=\sum_{i=1}^{N_{g}} \sum_{j=1}^{N_{g}} \left[ i+j-\mu_{x} \left( i \right)-\mu_{y} \left( j \right) \right]^{2}P(i,j)$$

(5) Contrast

$$contrast=\sum_{i=1}^{N_{g}} \sum_{j=1}^{N_{g}} \left| i-j \right|^{2}P(i,j)$$

(6) Correlation

$$correlation=\frac{\sum_{i=1}^{N_{g}} \sum_{j=1}^{N_{g}} ({i-\mu}_{x}){(j-\mu}_{y})P\left( i,j \right)}{\sigma_{x}\sigma_{y}}$$

(7) Difference average

$$difference average (\mu_{x-y})=\sum_{k=0}^{N_{g-1}} kp_{x-y}$$

(8) Difference entropy

$$difference entropy= -\sum_{i=0}^{N_{g-1}} p_{x-y}\left( i \right)\log_{2}[p_{x-y}(i)]$$

(9) Difference variance

$$difference variance= \sum_{i=0}^{N_{g-1}} {{(i-\mu_{x-y})}^{2}p}_{x-y}\left( i \right)$$

$$\mu_{x-y}=\frac{\sum_{k=0}^{N_{g-1}} \mathrm{His}\left( k \right)p_{x-y}}{\sum_{k=0}^{N_{g-1}} \mathrm{His}\left( k \right)}$$

His represents for histogram of abs(x-y).

(10) Inverse Difference

$$entropy=-\sum_{i=1}^{N_{g}} \sum_{j=1}^{N_{g}} P(i,j)\log_{2} \left[ P(i,j) \right]$$

(11) Homogeneity 1

$$homogeneity 1=\sum_{i=1}^{N_{g}} \sum_{j=1}^{N_{g}} \frac{P(i,j)}{1+\left| i-j \right|}$$

(12) Homogeneity 2

$$homogeneity 2=\sum_{i=1}^{N_{g}} \sum_{j=1}^{N_{g}} \frac{P(i,j)}{1+\left| i-j \right|^{2}}$$

(13) Informational measure of correlation 1 (IMC1)

$$IMC1=\frac{HXY-HXY1}{\max\left\{ HX,HY \right\}}$$

(14) Informational measure of correlation 2 (IMC2)

$$IMC2=\sqrt{1-e^{-2(HXY2-H)}}$$

(15) Inverse difference moment normalized (IDMN)

$$IDMN=\sum_{i=1}^{N_{g}} \sum_{j=1}^{N_{g}} \frac{p(i,j)}{1+(\frac{\left| i-j \right|^{2}}{N_{g}^{2}})}$$

(16) Inverse difference normalized (IDN)

$$IDN=\sum_{i=1}^{N_{g}} \sum_{j=1}^{N_{g}} \frac{p(i,j)}{1+(\frac{|i-j|}{N_{g}})}$$

(17) Inverse variance

$$inverse variance=\sum_{i=1}^{N_{g}} \sum_{j=1}^{N_{g}} \frac{P(i,j)}{\left| i-j \right|^{2}},i\neq j$$

(18) Maximal correlation coefficient

$$maximal correlation coefficient=\sqrt{second largest eigenvalue of Q}$$

$$Q=\sum_{k=1}^{N_{g}} \sum_{j=1}^{N_{g}} \frac{p\left( k,i \right)*p\left( j,i \right)}{p_{x}\left( k \right)*p_{y}}$$

(19) Maximum probability

$$maximum probability=max\left\{ P(i,j) \right\}$$

(20) Joint average

$$sum average=\sum_{i=1}^{Ng} \sum_{j=1}^{Ng} p(i,j)i$$

(21) Joint energy

$$sum entropy=-\sum_{i=2}^{{2N}_{g}} P_{x+y}(i)\log_{2} \left[ P_{x+y}(i) \right]$$

(22) Joint energy

$$joint energy=\sum_{i=1}^{Ng} \sum_{j=1}^{Ng} {(p(i,j))}^{2}$$

(23) Inverse difference moment (IDM)

$$IDM=\sum_{k=1}^{Ng-1} \left( \frac{p_{x-y}(k)}{1+k^{2}} \right)$$

2) GLRLM textural features (n=16)

GLRLM quantifies gray level runs in an image. A gray level run is defined as the length in number of pixels, of consecutive pixels that have the same gray level value. We used p(i, j| θ) to denote the GLRLM where the (i, j)^th^ element represents a pixel with a gray level i occurring consecutively at j times in 13 directions.

N_g_ is the number of discrete intensity levels in the image,

N_r_ is the number of different run lengths,

N_p_ is the number of voxels in the image,

$p_{n}(i,j)$ is the normalized run-length matrix, where $p_{n}\left( i,j \right)=\frac{p\left( i,j | \theta\right)}{\sum_{i=1}^{N_{g}} \sum_{j=1}^{N_{r}} p\left( i,j | \theta\right)}$,

$u_{g}$ is the mean gray level, where $u_{g}=\sum_{i=1}^{N_{g}} \sum_{j=1}^{N_{r}} ip_{n}(i,j)$,

$u_{r}$ is the mean run length, where $u_{r}=\sum_{i=1}^{N_{g}} \sum_{j=1}^{N_{r}} jp_{n}(i,j)$.

(1) Short run emphasis (SRE)

$$SRE=\frac{\sum_{i=1}^{N_{g}} \sum_{j=1}^{N_{r}} \left[ \frac{p(i,j|\theta)}{j^{2}} \right]}{\sum_{i=1}^{N_{g}} \sum_{j=1}^{N_{r}} p(i,j|\theta)}$$

(2) Long run emphasis (LRE)

$$LRE=\frac{\sum_{i=1}^{N_{g}} \sum_{j=1}^{N_{r}} j^{2}p(i,j|\theta)}{\sum_{i=1}^{N_{g}} \sum_{j=1}^{N_{r}} p(i,j|\theta)}$$

(3) Gray level non-uniformity (GLN)

$$GLN=\frac{\sum_{i=1}^{N_{g}} \left[ \sum_{j=1}^{N_{r}} p(i,j|\theta) \right]^{2}}{\sum_{i=1}^{N_{g}} \sum_{j=1}^{N_{r}} p(i,j|\theta)}$$

(4) Gray level non-uniformity normalized (GLNN)

$$GLNN=\frac{\sum_{i=1}^{N_{g}} {[\sum_{j=1}^{N_{r}} p(i,j|\theta)]}^{2}}{{[\sum_{i=1}^{N_{g}} \sum_{j=1}^{N_{r}} p\left( i,j | \theta\right)]}^{2}}$$

(5) Run length non-uniformity (RLN)

$$RLN=\frac{\sum_{j=1}^{N_{r}} \left[ \sum_{i=1}^{N_{g}} p(i,j|\theta) \right]^{2}}{\sum_{i=1}^{N_{g}} \sum_{j=1}^{N_{r}} p(i,j|\theta)}$$

(6) Run length non-uniformity normalized (RLNN)

$$RLN=\frac{\sum_{j=1}^{N_{r}} {[\sum_{i=1}^{N_{g}} p(i,j|\theta)]}^{2}}{{[\sum_{i=1}^{N_{g}} \sum_{j=1}^{N_{r}} p\left( i,j | \theta\right)]}^{2}}$$

(7) Run percentage (RP)

$$RP=\sum_{i=1}^{N_{g}} \sum_{j=1}^{N_{r}} \frac{p(i,j|\theta)}{N_{p}}$$

(8) Low gray level run emphasis (LGLRE)

$$LGLRE=\frac{\sum_{i=1}^{N_{g}} \sum_{j=1}^{N_{r}} \left[ \frac{p(i,j|\theta)}{i^{2}} \right]}{\sum_{i=1}^{N_{g}} \sum_{j=1}^{N_{r}} p(i,j|\theta)}$$

(9) High gray level run emphasis (HGLRE)

$$HGLRE=\frac{\sum_{i=1}^{N_{g}} \sum_{j=1}^{N_{r}} i^{2}p(i,j|\theta)}{\sum_{i=1}^{N_{g}} \sum_{j=1}^{N_{r}} p(i,j|\theta)}$$

(10) Short run low gray level emphasis (SRLGLE)

$$SRLGLE=\frac{\sum_{i=1}^{N_{g}} \sum_{j=1}^{N_{r}} \left[ \frac{p(i,j|\theta)}{i^{2}j^{2}} \right]}{\sum_{i=1}^{N_{g}} \sum_{j=1}^{N_{r}} [p(i,j|\theta)}$$

(11) Short run high gray level emphasis (SRHGLE)

$$SRHGLE=\frac{\sum_{i=1}^{N_{g}} \sum_{j=1}^{N_{r}} \left[ \frac{p(i,j|\theta)i^{2}}{j^{2}} \right]}{\sum_{i=1}^{N_{g}} \sum_{j=1}^{N_{r}} p(i,j|\theta)}$$

(12) Long run low gray level emphasis (LRLGLE)

$$LRLGLE=\frac{\sum_{i=1}^{N_{g}} \sum_{j=1}^{N_{r}} \left[ \frac{p(i,j|\theta)j^{2}}{i^{2}} \right]}{\sum_{i=1}^{N_{g}} \sum_{j=1}^{N_{r}} p(i,j|\theta)}$$

(13) Long run high gray level emphasis (LRHGLE)

$$LRHGLE=\frac{\sum_{i=1}^{N_{g}} \sum_{j=1}^{N_{r}} p(i,j|\theta)i^{2}j^{2}}{\sum_{i=1}^{N_{g}} \sum_{j=1}^{N_{r}} p(i,j|\theta)}$$

(14) Gray level variance (GLV)

$$GLV= \sum_{i=1}^{N_{g}} \sum_{j=1}^{N_{r}} \left( i-u_{g} \right)^{2}p_{n}(i,j)$$

(15) Run length variance (RLV)

$$RLV= \sum_{i=1}^{N_{g}} \sum_{j=1}^{N_{r}} \left( i-u_{r} \right)^{2}p_{n}(i,j)$$

(16) Run entropy (RE)

$$RE= \sum_{i=1}^{N_{g}} \sum_{j=1}^{N_{r}} p_{n}\left( i,j \right)\log_{2}[p_{n}(i,j)]$$

3) GLSZM (n=16)

GLSZM makes description of the amount of homogeneous connected areas within the volume, of a certain size and intensity[2]. The (i, j)^th^ entry of the GLDZM p(i, j) is the number of connected areas of gray-level i and size j.

$p(i,j)$is the (i,j)th entry in the given GLSZM p,

$N_{g}$ is the number of discrete intensity values in the image,

$N_{Z}$ is the size of the largest, homogeneous region in the volume of interest

$N_{s}$ is the total number of homogeneous regions (zones), where $N_{s}=\sum_{i=1}^{N_{g}} \sum_{j=1}^{N_{d}} p(i,j)$,

$p_{z}$ is the sum distribution of the number of zones with size $j$, where $p_{z}(j)=\sum_{i=1}^{N_{g}} p(i,j)$,

$p_{g}$ is the sum distribution of the number of zones with gray-level i, where $p_{g}\left( i \right)=\sum_{j=1}^{N_{d}} p\left( i,j \right)$,

$N_{p}$ is the number of voxels in the image, where $N_{p}=\sum_{j=1}^{N_{d}} jp_{z}$,

$p_{n}(i,j)$ is the normalized size-zone matrix, where $p_{n}\left( i,j \right)=\frac{p(i,j)}{N_{s}}$,

$u_{z}$ is the mean size, where $u_{z}=\sum_{i=1}^{N_{g}} \sum_{j=1}^{N_{d}} jp_{n}(i,j)/(N_{g}*N_{d})$,

$u_{g}$ the mean gray level, where $u_{g}=\sum_{i=1}^{N_{g}} \sum_{j=1}^{N_{d}} ip_{n}(i,j)/(N_{g}*N_{d})$.

(1) Small area emphasis (SAE)

$$SAE= \frac{1}{N_{s}}\sum_{j} \frac{p_{z}}{j^{2}}$$

(2) Large area emphasis (LAE)

$$LAE= \frac{1}{N_{s}}\sum_{j} j^{2}p_{z}$$

(3) Intensity Non-uniformity (IN)

$$IN= \frac{1}{N_{s}^{2}}\sum_{i} p_{g}^{2}$$

(4) Intensity Non-uniformity Normalized (INN)

$$INN= \frac{1}{N_{s}^{3}}\sum_{i} p_{g}^{2}$$

(5) Size-zone Non-uniformity (SZN)

$$SZN= \frac{1}{N_{s}^{2}}\sum_{j} p_{z}^{2}$$

(6) Size-zone Non-uniformity normalized (SZNN)

$$SZNN= \frac{1}{N_{s}^{3}}\sum_{j} p_{d}^{2}$$

(7) Zone percentage (ZP)

$$ZP=\frac{{N_{s}}^{3}}{N_{p}}$$

(8) Low intensity emphasis (LIE)

$$LIE= \frac{1}{N_{s}}\sum_{i} \frac{p_{g}}{i^{2}}$$

(9) High intensity emphasis (HIE)

$$HIE= \frac{1}{N_{s}}\sum_{i} i^{2}p_{g}$$

(10) Low intensity small area emphasis (LISAE)

$$LISAE= \frac{1}{N_{s}}\sum_{i} \sum_{j} \frac{p(i,j)}{i^{2}j^{2}}$$

(11) High intensity small area emphasis (HISAE)

$$HISAE= \frac{1}{N_{s}}\sum_{i} \sum_{j} \frac{p(i,j)i^{2}}{j^{2}}$$

(12) Low intensity large area emphasis (LILAE)

$$LILAE= \frac{1}{N_{s}}\sum_{i} \sum_{j} \frac{p(i,j)j^{2}}{i^{2}}$$

(13) High intensity large area emphasis (HILAE)

$$HILAE= \frac{1}{N_{s}}\sum_{i} \sum_{j} p(i,j)i^{2}j^{2}$$

(14) Intensity variance (IV)

$$IV=\frac{1}{N_{s}}\sum_{i} \sum_{j} \left( i-u_{g} \right)^{2}p_{n}\left( I,j \right)$$

(15) Size-zone variance (SZV)

$$SZV=\frac{1}{N_{s}}\sum_{i} \sum_{j} \left( j-u_{d} \right)^{2}p_{n}\left( i,j \right)$$

(16) Zone entropy (ZE)

$$ZE=\sum_{i} \sum_{j} p_{n}\left( i,j \right)\log_{2}[p_{n}\left( i,j \right)]$$

4) NGLDM (n = 16)

NGLM features are invariant under spatial rotation [3]. The (i, j)^th^ entry of the NGLDM p(i, j | d, a) depicts the number of neighborhoods with center voxel gray level i and dependence k = j – 1. A neighborhood are all voxels with a distance d from the center voxel. The center voxel and a neighboring voxel are dependent if their absolute gray value difference $\leq$ a, the dependency coarseness parameter.

$p\left( i,j \right)$ is the (i,j)th entry in the given NGLDM p, generalized for any d and a,

$N_{g}$ is the number of discrete intensity values in the image,

$N_{d}$ is the maximum dependence value,

$N_{s}$ is the total number of neighborhoods, where $N_{s}=\sum_{i=1}^{N_{g}} \sum_{j=1}^{N_{d}} p(i,j)$,

$p_{d}$ is the sum distribution of the number of neighborhoods with dependence $j=k+1$, where $p_{d}(j)=\sum_{i=1}^{N_{g}} p(i,j)$,

$p_{g}$ is the sum distribution of the number of neighborhoods with center voxel gray level $i$, where $p_{g}\left( i \right)=\sum_{j=1}^{N_{d}} p\left( i,j \right)$,

$p_{n}(i,j)$ is the normalized NGLDM, where $p_{n}\left( i,j \right)=\frac{p(i,j)}{N_{s}}$,

$u_{d}$ is the mean dependence, where $u_{d}=\sum_{i=1}^{N_{g}} \sum_{j=1}^{N_{d}} jp_{n}(i,j)/\left( N_{g}*N_{d} \right)$,

$u_{g}$ is the mean gray level, where $u_{g}=\sum_{i=1}^{N_{g}} \sum_{j=1}^{N_{d}} ip_{n}(i,j)/(N_{g}*N_{d})$.

(1) Small distance emphasis (SDE)

$$SDE= \frac{1}{N_{s}}\sum_{j} \frac{p_{d}}{j^{2}}$$

(2) Large distance emphasis (LDE)

$$LDE= \frac{1}{N_{s}}\sum_{j} j^{2}p_{d}$$

(3) Gray-level Non-uniformity (GLN)

$$GLN= \frac{1}{N_{s}^{2}}\sum_{j} p_{d}^{2}$$

(4) Gray-level Non-uniformity Normalized (GLNN)

$$GLNN= \frac{1}{N_{s}^{3}}\sum_{j} p_{d}^{2}$$

(5) Dependence Non-uniformity (DN)

$$DN= \frac{1}{{N_{s}}^{2}}\sum_{i} p_{g}^{2}$$

(6) Dependence Non-uniformity normalized (DNN)

$$DNN=\frac{1}{N_{s}^{3}}\sum_{i} p_{g}^{2}$$

(7) Low gray-level emphasis (LGE)

$$LGE= \frac{1}{N_{s}}\sum_{i} \frac{p_{g}}{i^{2}}$$

(8) High gray-level emphasis (HGE)

$$HGE= \frac{1}{N_{s}}\sum_{i} i^{2}p_{g}$$

(9) Low gray-level small dependence emphasis (LGSDE)

$$LGSDE= \frac{1}{N_{s}}\sum_{i} \sum_{j} \frac{p(i,j)}{i^{2}j^{2}}$$

(10) High gray-level small dependence emphasis (HGSDE)

$$HGSDE= \frac{1}{N_{s}}\sum_{i} \sum_{j} \frac{p(i,j)i^{2}}{j^{2}}$$

(11) Low gray-level large dependence emphasis (LGLDE)

$$LGLDE= \frac{1}{N_{s}}\sum_{i} \sum_{j} \frac{p(i,j)j^{2}}{i^{2}}$$

(12) High gray-level large dependence emphasis (HGLDE)

$$HGLDE= \frac{1}{N_{s}}\sum_{i} \sum_{j} p(i,j)i^{2}j^{2}$$

(13) Gray-level variance (GLV)

$$GLV=\frac{1}{N_{s}}\sum_{i} \sum_{j} \frac{\left( i-u_{g} \right)^{2}p_{n}\left( i,j \right)}{N_{g}*N_{d}}$$

(14) Dependence variance (DV)

$$DZV=\frac{1}{N_{s}}\sum_{i} \sum_{j} \frac{\left( j-u_{d} \right)^{2}p_{n}\left( i,j \right)}{N_{g}*N_{d}}$$

(15) Dependence entropy (DE)

$$DE=N_{s}*\sum_{i} \sum_{j} p_{n}\left( i,j \right)\log_{2}[p_{n}\left( i,j \right)]$$

(16) Second moment (SM)

$$SM= \frac{\sum_{i} \sum_{j} {p(i,j)}^{2}}{N_{s}^{2}}$$

5) NGTDM (n = 5)

NGTDM extracts features based on the visual properties of the tumor region [4]. It is a column matrix formed by the absolute value of the pixel being observed minus the average of the pixels in its neighborhood. There are 5 NGTDM features in total. Let f(k, l) be the gray tone of any pixel at (k, l) having gray tone value i. Then it finds the average gray-tone over a neighborhood centered at, but excluding (k, l)

$$\bar{A_{i}}= \bar{A}\left( k, l \right)= \frac{1}{W-1}[\sum_{m= -d}^{d} \int_{n= -d}^{d} f(k+m, l+n)]$$

where d specifies the neighborhood size and W = (2d+1)^2^.

Then the i^th^ entry in the NGTDM is

$S\left( i \right)= \sum\left| i-\bar{A_{i}} \right|, for i\in N_{i}\mathrm{if}N_{i} \neq0$

$= 0, otherwise$

Where N_i_ is the set of all pixels having gray tone I (except in the peripheral regions of width d).

(1) Coarseness

$$coarseness=\frac{I}{\varepsilon+\sum_{i} p(i)S(i)}$$

where $\varepsilon$ is the small number to prevent coarseness becoming infinite, p(i) is the probability of occurrence of gray-tone level i.

(2) Contrast

$$contrast=\left( \frac{1}{N_{p}(N_{p}-1)}\sum_{i} \sum_{j} p(i)p(j){(i-j)}^{2} \right)\left( \frac{1}{n}\sum_{i} S(i) \right)$$

where,

for an N $\times$ N image, n = N $-$ 2d,

N_p_ =$\sum_{i} Q_{i}$, where Q_i_ = $\left\{ \begin{aligned} 1, if p(i)\neq0 \\ 0, otherwise \end{aligned} \right.$.

(3) Busyness

$$Busyness=\frac{\sum_{i} (p(i)S(i))}{\sum_{i} \sum_{j} |ip(i)-jp(j)|}$$

(4) Complexity

$complexity=\frac{1}{n}\sum_{i} \sum_{j} |i-j|\frac{(p(i)S(i)+p(j)S(j)}{p\left( i \right)+p(j)}$,

(5) Strength

$$strength=\frac{\sum_{i} \sum_{j} [p\left( i \right)+p\left( j \right)]\left( i-j \right)^{2}}{\varepsilon+\sum_{i} S(i)}$$

**E5: Multiple Segmentation for feature robustness analysis**

To perform feature robustness analysis, we randomly selected 20 patients and applied three kinds of multiple segmentation: multiple clinician segmentation, multiple time-point segmentation, and perturbation segmentation.

(1) Multiple Clinician Segmentation

For each patient, two counterpart radiologists segmented the tumor lesion in a double blinded manner at the same time.

(2) Multiple Time-Point Segmentation

For each patient, test-retest variability was measured through two times segmentation by the same radiologist with time interval of three days.

(3) Morphological Perturbation Segmentation

We performed morphological perturbation on the boundaries in 8 manners as follows:

1. Left shifting by 3 pixels
2. Right shifting by 3 pixels
3. Up shifting by 3 pixels
4. Down shifting by 3 pixels
5. Morphologically opening with 3🞨 3 square window
6. Morphologically closing with 3 🞨 3 square window
7. Clockwise rotation by 3°
8. Counter clockwise rotation by 3 °

**E6: Description of 16 feature selection and 4 classification algorithms**

We hereby implemented 16 feature selection algorithms as recommended in [5]. We chose scikit-learn as the effective software package through coding language Python. A brief introduction of the16 feature selection algorithms are as follows:

(1) Recursive Feature Elimination (RFE)

RFE was proposed by Guyon et al [6]. It aims to rank the feature set and recursively remove the ones contributing least to the classification. The estimator is primarily trained by the initial feature set and weights are assigned. Features with smallest weight would be pruned from the current feature set. This procedure will be repeated until desired feature number was achieved.

(2) Minimum Redundancy Maximum Relevance (MRMR)

MRMR selects top ranking features based on relevancy-redundancy tradeoff [7]. Features that are mutually far away from each other, meanwhile, highly correlated with the classification labels were remained. Let I(X_k_, Y) be the mutual information between the feature X_k_ and the classification labels, and S be the set of features. The scoring criterion is defined as：

J_mrmr_(X_k_) = I(X_k_; Y) – $\frac{1}{|S|}\sum_{X_{j}\epsilon S} I(X_{k};X_{j})$

(3) Interaction Capping (ICAP)

ICAP selects features with strong pairwise conditional dependence with the already selected feature set S and maximum mutual relevancy with the classification labels [8]. The scoring criterion is defined as:

J_icap_(X_k_) = I(X_k_; Y) – $\sum_{X_{j}\epsilon S} max[0, \left\{ I\left( X_{k};X_{j} \right)-I(X_{k};X_{j}|Y) \right\}]$

(4) Mutual Information Maximization (MIM)

MIM measures the relevance of the feature and the classification labels based on information theory [9]. The scoring criterion is defined as:

J_mim_(X_k_) = I(X_k_; Y)

(5) Conditional Mutual Information Maximization (CMIM)

CMIM selects features with maximum mutual information with the classification labels conditioned on the feature set S is already existed [10]. The scoring criterion is defined as:

J_cmim_(X_k_) =$\min_{X_{j}\epsilon S}$ [I(X_k_; Y|X_j_)]

(6) Conditional Infomax Feature Extraction (CIFE)

CIFE aims to select correlated features with strong class conditional dependence [11]. The scoring criterion is defined as:

J_cife_(X_k_) = I(X_k_; Y) – [$I\left( X_{k};X_{j} \right)-I(X_{k};X_{j}|Y)$]

(7) Joint Mutual Information (JMI)

Similar to MIM, JMI introduces a joint random variable X_k_X_j_ [12]. the scoring criterion is defined as:

$$J_{\mathrm{jmi}}\left( X_{k} \right)=\sum_{X_{j}\epsilon S} I(X_{k}X_{j};Y)$$

(8) Double Input Symmetric Relevance (DISR)

DISR accepts the modification of joint mutual information criterion [13]. A joint entropy term was introduced to build the joint mutual information. The scoring criterion is defined as:

$$J_{\mathrm{disr}}\left( X_{k} \right)=\sum_{X_{j}\epsilon S} \frac{I(X_{k}X_{j};Y)}{H(X_{k}X_{j}Y)}$$

(9) Mutual Information Feature Selection (MIFS)

MIFS is similar to MRMR, it aims to select feature with relevance to the classification labels and least feature redundancy [14]. Let $\beta$ be the configurable parameter, which should be set experimentally. The scoring criterion is defined as:

$$J_{\mathrm{mifs}}\left( X_{k} \right)=I\left( X_{k};Y \right)-\beta\sum_{X_{j}\epsilon S} I(X_{k};X_{j})$$

(10) Trace Ratio

Trace ratio criterion is proposed based on linear discriminant analysis (LDA) which aims to find the optimal low-dimensional manner to maximize the between-class scatter matrix and minimize the within-class scatter matrix. LDA only works with binary-class classification. When it comes to multi-class classification problem, trace ratio criterion exists. It reflects the Euclidean distances between data of inter- and intra-classes. The optimal projection obtained by trace ratio LDA is orthogonal, which may reserve similarity. Detailed prediction of Trace ratio is shown in [15].

(11) Fisher Score (FSCR)

FSCR selects that have minimized distance within the same class and maximized distance between the classes [16]. Let $\mu$_k_ be the overall mean of feature X_k_, m be the number of cases in the m^th^ class, and $\mu$_k,m_ and $\sigma$_k,m_ be the mean and variabce of feature X_k_ in m^th^ class. The scoring criterion is defined as follows:

$$J_{\mathrm{fisher}}\left( X_{k} \right)=\frac{\sum_{m=1}^{2} n_{m}{(\mu_{k,m} - \mu_{k})}^{2}}{\sum_{m=1}^{2} n_{m}\sigma_{k,m}^{2}}$$

(12) Spectral Feature Selection (SPEC)

As descript in [17], spectral feature selection is based on graph theory. It employs the spectrum of the graph to measure feature relevance. If a feature assigns values to the instance consistently with the graph structure, which means it can separate the data better, thus it will be remained in the selected feature set.

(13) Correlation-based Feature Selection (CFS)

CFS selects features based on correlation heuristic to calculate the worth of features [18]. Let k be the feature number in feature set S, R(X, Y) = $\sum_{X_{j}\epsilon S} \frac{1}{k}\sum(X_{j}, Y)$ be the mean correlation between features in feature set S and the classification labels, and R(X, X) be the mean correlation between features in feature set S. The merit of the feature is defined as:

Merit_S_ = $\frac{kR(X,Y)}{\sqrt{k+k(k-1)R(X,X)}}$

(14) Gini Index

Let p(m|X_k_) be the conditional probability of m^th^ class when feature X_k_ is given [19]. The scoring criterion is defined as:

$$J_{\mathrm{gini}}\left( X_{k} \right)=1-(\sum_{m=1}^{2} {[p(m\left| X_{k} \right.)]}^{2})$$

Features are selected with larger gini index values with correspondence to lower feature relevance.

(15) Robust Feature Selection (RFS)

RFS was designed to employ joint l_2,1_-norm minimization on both loss function and regularization. l_2,1_-norm based loss function is adopted to remove outliers, and l_2,1_-norm regularization is performed to select features with joint sparsity. Detailed description of RFS is shown in [20].

(16) Multi-Cluster Feature Selection (MCFS)

MCFS is used for unsupervised feature selection based on abovementioned SPEC and L1-regularized models for subset selection. It aims to select features to preserve multi-cluster structure of the data. Detailed description of MCFS is shown in [21].

Referring to the optimal classifier, after statistics, 4 most commonly used algorithms in radiomics studies were found to be logistic regression (LR), decision tree (DT), random forest (RF), and support vector machine (SVM). A brief introduction of these three classifiers are as follows:

(1) Logistic Regression

LR was proposed by statistician David Cox in 1958 [22]. It is an appropriate regression analysis when the dependent variable is binary. It is used to depict the relationship between one dependent binary variable and one or more independent predictors. Mathematically, logistic regression estimates a multiple linear regression function as follows:

logit(p) = log$\frac{p}{1-p}$ = b_0_ + b_1_ X_1_ + b_2_ X_2_ + b_3_ X_3_ + … + b_k_ X_k_

where p is the possibility of presence of the event, X is the independent variables.

(2) Decision Tree

Decision tree realize classification through step-by-step feature selection. From the bottom of the tree, maximize the information gain function of each node in each layer. The information gain function is as follow:

$G\left( \emptyset\right)=H\left( Q \right)-\sum_{S\in\left\{ l,r \right\}} \frac{\left| Q_{S}\left( \emptyset\right) \right|}{\left| Q \right|}H\left( Q_{S}\left( \emptyset\right) \right)$

$\emptyset$ represents for model parameters, $G\left( \emptyset\right)$ represents for the information gain, $H\left( Q \right)$represents for entropy, $S\in\left\{ l,r \right\}$represents the split from left tree to the right tree. The tree stops growing until each node only contains one class.

(3) Random Forest

RF builds a meta estimator with multiple decision trees based on subgroups of the dataset [23]. It applies the average result of each component decision tree to improve the predictability of the model. Generally speaking, RF performed much better than the single decision tree and has the ability to control overfitting.

(4) Support Vector Machine

SVM constructs a hyperplane in high dimensional space that separates different classes [24]. Larger functional margin represents for better separation by the hyperplane, which means the generalization error is lower. Both regression and classification tasks could be realized through SVM. It can dispose continuous and categorical variables.

**E7: User instruction for CRIM online tool**

##### Implemented by Jingwei Wei, Yuqi Han, and Dongsheng Gu. Please contact [weijingwei2014@ia.ac.cn](mailto:weijingwei2014@ia.ac.cn) or [dongshegngu2016@ia.ac.cn](mailto:dongshegngu2016@ia.ac.cn), if you have any question.

This code is packaged and released. Users can unzip HPC_predict.zip file, and double click ‘HPC_model.exe’ in folder path “exe.win-amd64-3.5” to run the program.

**Instructions:**

1. Double click ‘HPC_model.exe’.

2. Click ‘browse’ button, and input the original image folder path “T1 dicom file path”, “T1-EN dicom file path”, “T2 dicom file path”, segmentation mask folder path “Tumor Segment file path”, and clinical table file path “Clinic info file path”. An example of clinical table, see download file “testpatient.csv”.
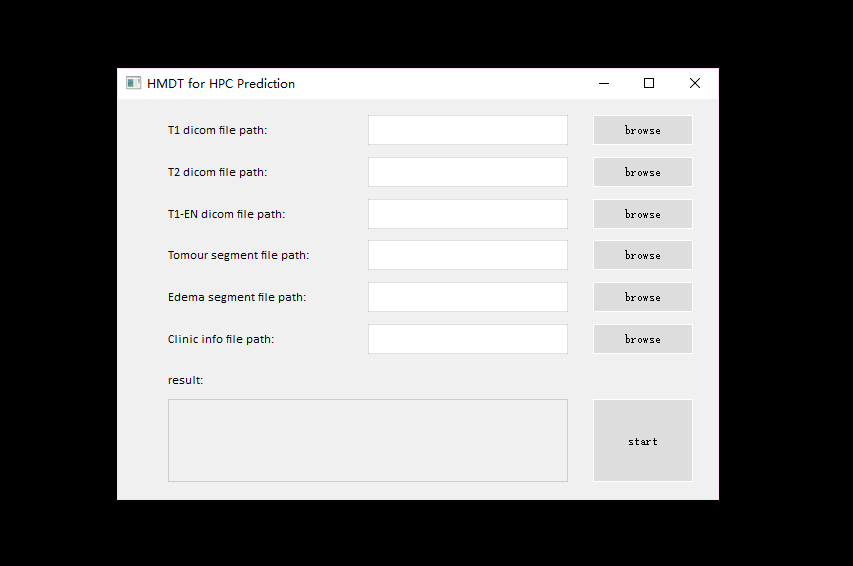


3. After inputting the file paths, click ‘start’ button. Wait for less than 30-40 seconds, the predicted diagnosis outcome of the patient can be acquired, including probability of HPC, diagnosis outcome, and the cut-off value of the nomogram.


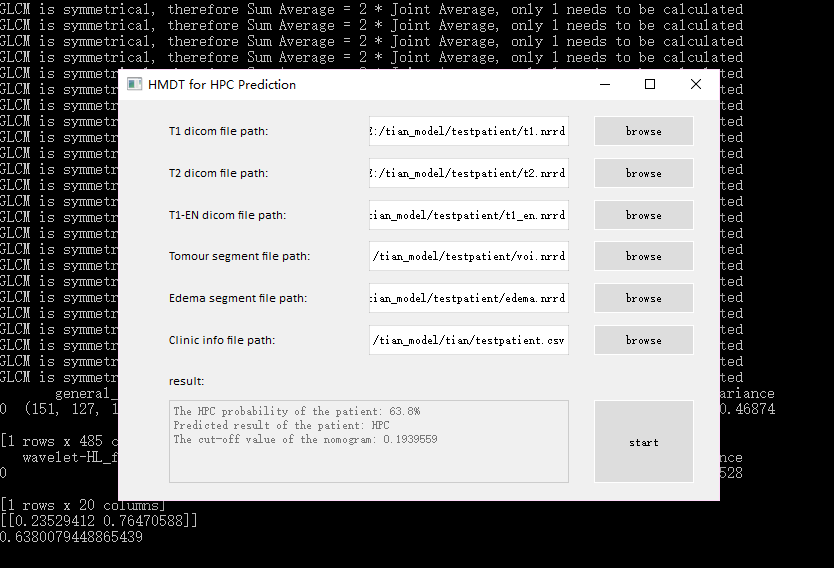


**Note:**

1) The original image format should be medical imaging format ‘.dicom’.

2) The segmentation mask format should be medical imaging format ‘.nrrd’. Users could implement segmentation by ITK-SNAP, 3D slicer, etc.

3) Clinical variable only conclude dural tail sign, 0 represents for no dural tail sign; 1 represents for dural tail sign existing. Clinical table example can be acquired in the download file ‘testpatient.csv’.

4) If it reports error when opening the program, please close antivirus software first.

5) It may cost 1-2 minutes to open the program for the first time use.

This software is for ACADEMIC USE only.

Please cite article “Multi-habitat and multi-sequence based radiomics for preoperative and noninvasive diagnosis of intracranial hemangiopericytomas from meningiomas”, if using the model and program.

**E8: Supplementary Figures**


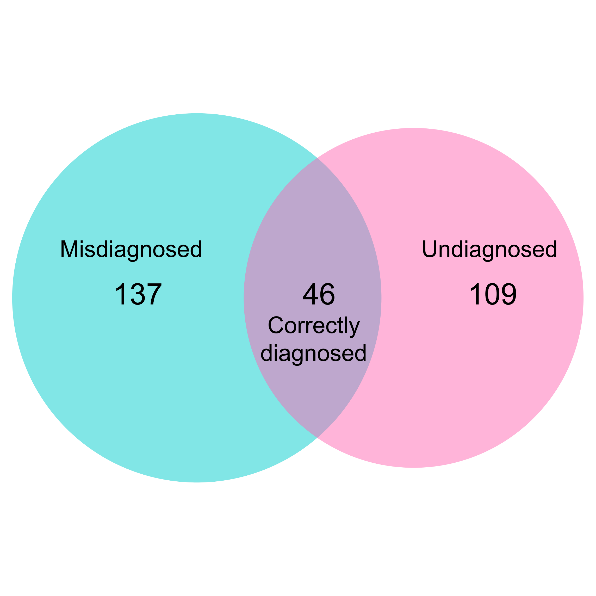


**Supplementary Figure 1. Preoperative diagnosis of IHPC and meningioma** 292 patients were enrolled in the present study. 46 cases were correctly diagnosed. 137 pathologically confirmed meningiomas were radiologically misdiagnosed as IHPC and 109 pathologically confirmed IHPC were radiologically undiagnosed.

**
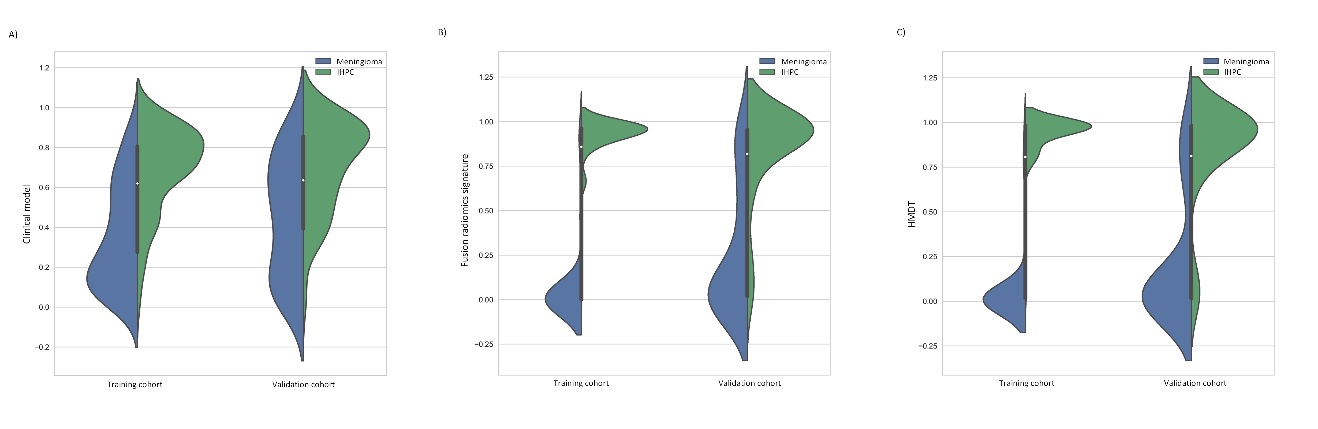
Supplementary Figure 2. Violin graphs.** The violin graphs demonstrated the value distribution of clinical model, fusion radiomics signature, and HMDT were shown in subfigures A, B, and C, respectively.

**
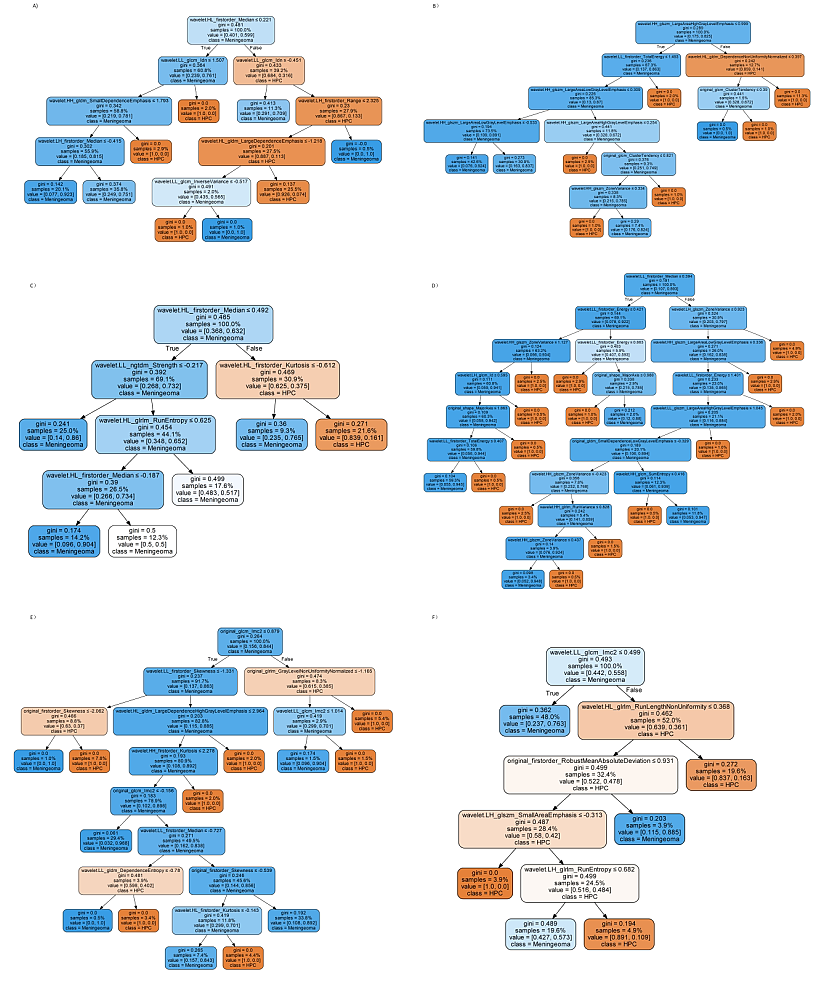
Supplementary Figure 3. Decision trees.** The corresponding decision trees for T1WI-tumor, T1WI-edema, CE-T1WI-tumor, CE-T1WI-edema, T2WI-tumor, T2WI-edema, respectively.

**
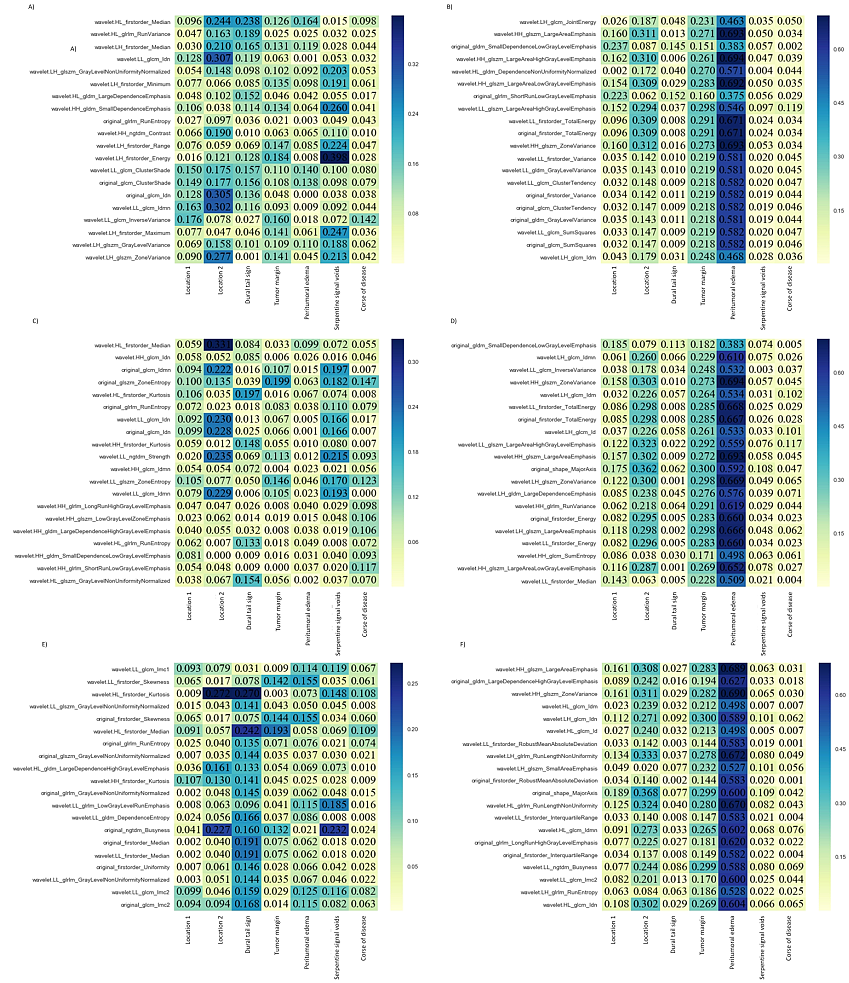
Supplementary Figure 4.** The heat maps revealed the correlation between the selected clinical factors and the selected 20 radiomic features for each single radiomics signature. The radiomics signature were signature^T1WI-tumor^, signature^CE-T1WI-tumor^, signature^T2WI-tumor^, signature^T1WI-edema^, signature^CE-T1WI-edema^, and signature^T2WI-edema^, and the corresponding subfigures were A-F. Statistical significance was defined with a two-sided p-value less than 0.05.

**E9: Supplementary Tables**

**Supplementary Table 1. Uni- and multi- variable analysis of clinic-radiological factors**

|  | **Uni-variable analysis** | | **Multi-variable analysis** | | **AUC**  **(95% CI)** | |
| --- | --- | --- | --- | --- | --- | --- |
| **Factor** | **p-value** | **OR**  **(95% CI)** | **p-value** | **OR**  **(95%CI)** | **Training cohort**  **(n=204)** | **Validation cohort**  **(n=88)** |
| **Age** | 0.004 | 0.970  (0.950-0.990) | 0.209 | 0.984  (0.960-1.009) |  |  |
| **Gender** | 0.471 | 1.225  (0.706-2.125) |  |  |  |  |
| **Course of disease** | 0.078 | 0.989  (0.977-1.001) | 0.030* | 0.984 (0.970-0.998) | 0.518  (0.437-0.600) | 0.522  (0.398-0.647) |
| **Location 1** | <0.001 | 0.328  (0.285-0.583) | 0.016* | 0.426  (0.213-0.853) | 0.635  (0.569-0.701) | 0.502  (0.397-0.607) |
| **Location 2** | 0.002 | 0.227  (0.089-0.580) | 0.043* | 0.284  (0.084-0.963) | 0.417  (0.370-0.464) | 0.392  (0.326-0.459) |
| **Location 3** | 0.084 | 1.362  (0.959-1.935) | 0.948 | 0.985  (0.627-1.548) |  |  |
| **Midline type** | 0.276 | 1.401  (0.764-2.571) |  |  |  |  |
| **Venous sinus invasion** | 0.519 | 0.834  (0.480-1.448) |  |  |  |  |
| **Dural tail sign** | <0.001 | 0.164  (0.086-0.312) | <0.001* | 0.162  (0.075-0.354) | 0.691  (0.630-0.752) | 0.340  (0.252-0.428) |
| **Tumor shape** | 0.410 | 0.803  (0.476-1.354) |  |  |  |  |
| **Enhancement pattern** | 0.611 | 1.190  (0.608-2.332) |  |  |  |  |
| **Tumor margin** | 0.020 | 0.464  (0.243-0.886) | 0.003* | 0.268  (0.113-0.636) | 0.429  (0.369-0.488) | 0.429  (0.332-0.526) |
| **Peritumoral edema** | 0.004 | 0.473  (0.283-0.785) | 0.006* | 0.385  (0.196-0.757) | 0.400  (0.335-0.565) | 0.403  (0.297-0.510) |
| **Serpentine signal voids** | 0.004 | 2.932  (1.412-6.090) | 0.019* | 3.150  (1.212-8.188) | 0.583  (0.528-0.637) | 0.590  (0.503-0.676) |

**Note:** OR: odds ratio; CI: confidence interval; AUC: area under curve; Location 1: frontal/posterior; Location 2: supra/infra; Location 3: left/right/both; *P < 0.05.

**Supplementary Table 2. Remained feature numbers after stability, reproducibility, and relevance analysis for each sequence and habitat**

| **Sequence and habitat** | **CCC** | **ICC** | **p-value** |
| --- | --- | --- | --- |
| **T1_tumor** | 463 | 446 | 201 |
| **T2_tumor** | 465 | 438 | 120 |
| **CE-T1_tumor** | 452 | 439 | 24 |
| **T1_edema** | 375 | 311 | 167 |
| **T2_edema** | 391 | 287 | 156 |
| **CE-T2_edema** | 229 | 364 | 109 |

**Note:** CCC: concordance correlation coefficient; ICC: intra-class correlation coefficient. P-value was calculated by Mann-Whitney U test.

**Supplementary Table 3. Final selected features for each sequence and habitat**

| **T1_tumor** | | | | | |
| --- | --- | --- | --- | --- | --- |
| **Number** | **Radiomic feature** | **p-value** | **AUC (95% CI)** | | **Description** |
|  |  |  | **Training cohort**  **(n=204)** | **Validation cohort**  **(n=88)** |  |
| 1 | wavelet.HL_firstorder_Median | <0.001 | 0.725  (0.655-0.796) | 0.835  (0.748-0.922) | The median gray level intensity within the image transformed by wavelet filter XHL. |
| 2 | wavelet.HL_glrlm_RunVariance | 0.009 | 0.606  (0.528-0.683) | 0.537  (0.410-0.665) | RunVariance is a measure of the variance in runs for the run lengths of the image transformed by wavelet filter XHL. |
| 3 | wavelet.LH_firstorder_Median | <0.001 | 0.723  (0.653-0.792) | 0.798  (0.703-0.892) | The median gray level intensity within the image transformed by wavelet filter XLH. |
| 4 | wavelet.LL_glcm_Idn | <0.001 | 0.677  (0.603-0.751) | 0.577  (0.56-0.698) | IDN is a measure of the local homogeneity of the image transformed by wavelet filter XLL. |
| 5 | wavelet.LH_glszm_GrayLevelNonUniformityNormalized | 0.002 | 0.624  (0.547-0.702) | 0.688  (0.574-0.802) | GLNN measures the similarity of gray-level intensity values in the image transformed by wavelet filter XLH. |
| 6 | wavelet.LH_firstorder_Minimum | 0.020 | 0.594  (0.516-0.672) | 0.697  (0.586-0.808) | The minimum gray level intensity within the image transformed by wavelet filter XLH. |
| 7 | wavelet.HL_gldm_LargeDependenceEmphasis | 0.049 | 0.579  (0.502-0.658) | 0.547  (0.418-0.675) | LDE is a measure of the distribution of large dependencies, with a greater value indicative of larger dependence and more homogeneous textures. |
| 8 | wavelet.HH_gldm_SmallDependenceEmphasis | 0.036 | 0.585  (0.505-0.665) | 0.607  (0.488-0.726) | SDN is a measure of the distribution of small dependencies, with a greater value indicative of smaller dependence and less homogeneous textures. |
| 9 | original_glrlm_RunEntropy | 0.003 | 0.622  (0.543-0.700) | 0.589  (0.468-0.710) | RE measures the uncertainty/randomness in the distribution of run lengths and gray levels the original image. |
| 10 | wavelet.HH_ngtdm_Contrast | 0.026 | 0.590  (0.511-0.669) | 0.753  (0.649-0.857) | Contrast is a measure of the spatial intensity change of the image transformed by wavelet filter XHH. |
| 11 | wavelet.LH_firstorder_Range | 0.024 | 0.592  (0.514-0.670) | 0.699  (0.589-0.810) | The range of gray values in the image transformed by wavelet filter XLH. |
| 12 | wavelet.LH_firstorder_Energy | 0.030 | 0.588  (0.510-0.666) | 0.584  (0.464-0.704) | Energy is a measure of the magnitude of voxel values in the image transformed by wavelet filter XLH. |
| 13 | wavelet.LL_glcm_ClusterShade | < 0.001 | 0.694  (0.622-0.766) | 0.655  (0.537-0.773) | Cluster Shade is a measure of the skewness and uniformity of the GLCM of the image transformed by wavelet filter XLL. |
| 14 | original_glcm_ClusterShade | < 0.001 | 0.694  (0.622-0.766) | 0.654  (0.536-0.772) | Cluster Shade is a measure of the skewness and uniformity of the GLCM of the original image. |
| 15 | original_glcm_Idn | < 0.001 | 0.681  (0.608-0.755) | 0.432  (0.311-0.554) | IDN is a measure of the local homogeneity of the original image. |
| 16 | wavelet.LL_glcm_Idmn | < 0.001 | 0.660  (0.585-0.735) | 0.554  (0.433-0.676) | IDMN is a measure of the local homogeneity of the image transformed by wavelet filter XLL. |
| 17 | wavelet.LL_glcm_InverseVariance | 0.025 | 0.591  (0.512-0.670) | 0.573  (0.450-0.696) | ID is a measure of the local homogeneity of the image transformed by wavelet filter XLL. |
| 18 | wavelet.LH_firstorder_Maximum | 0.044 | 0.582  (0.503-0.661) | 0.675  (0.561-0.789) | The maximum gray level intensity within the image transformed by wavelet filter XLH. |
| 19 | wavelet.LH_glszm_GrayLevelVariance | 0.002 | 0.626  (0.549-0.704) | 0.701  (0.590-0.813) | GLV measures the variance in gray level intensities for the zones of the image transformed by wavelet filter XLH. |
| 20 | wavelet.LH_glszm_ZoneVariance | 0.046 | 0.581  (0.502-0.660) | 0.605  (0.486-0.723) | ZV measures the variance in zone size volumes for the zones of the image transformed by wavelet filter XLH. |
| **CE-T1_tumor** | | | | | |
| **Number** | **Radiomic feature** | **p-value** | **AUC (95% CI)** | | **Description** |
|  |  |  | **Training cohort**  **(n=204)** | **Validation cohort**  **(n=88)** |  |
| 1 | wavelet.HL_firstorder_Median | < 0.001 | 0.640  (0.562-0.718) | 0.702  (0.591-0.814) | The median gray level intensity within the image transformed by wavelet filter XHL. |
| 2 | wavelet.HH_glcm_Idn | 0.012 | 0.602  (0.524-0.680) | 0.615  (0.495-0.735) | IDN is a measure of the local homogeneity of the image transformed by wavelet filter XHL. |
| 3 | original_glcm_Idmn | 0.041 | 0.583  (0.504-0.662) | 0.520  (0.397-0.644) | IDMN is a measure of the local homogeneity of the original image. |
| 4 | original_glszm_ZoneEntropy | 0.007 | 0.610  (0.532-0.688) | 0.570  (0.448-0.692) | ZE measures the uncertainty/randomness in the distribution of zone sizes and gray levels in the original image. |
| 5 | wavelet.HL_firstorder_Kurtosis | 0.008 | 0.608  (0.531-0.686) | 0.525  (0.402-0.648) | Kurtosis is a measure of the ‘peakedness’ of the distribution of values in the image transformed by wavelet filter XHL. |
| 6 | original_glrlm_RunEntropy | 0.035 | 0.585  (0.506-0.665) | 0.540  (0.415-0.665) | RE measures the uncertainty/randomness in the distribution of run lengths and gray levels the original image. |
| 7 | wavelet.LL_glcm_Idn | 0.004 | 0.618  (0.541-0.696) | 0.500  (0.377-0.623) | IDN is a measure of the local homogeneity of the image transformed by wavelet filter XLL. |
| 8 | original_glcm_Idn | 0.004 | 0.616  (0.538-0.694) | 0.512  (0.389-0.636) | IDN is a measure of the local homogeneity of the original image. |
| 9 | wavelet.HH_firstorder_Kurtosis | 0.017 | 0.597  (0.518-0.675) | 0.659  (0.544-0.775) | Kurtosis is a measure of the ‘peakedness’ of the distribution of values in the image transformed by wavelet filter XHH. |
| 10 | wavelet.LL_ngtdm_Strength | 0.013 | 0.561  (0.482-0.641) | 0.583  (0.462-0.704) | Strength is a measure of the primitives in the image transformed by wavelet filter XLL. |
| 11 | wavelet.HH_glcm_Idmn | 0.015 | 0.599  (0.521-0.677) | 0.615  (0.495-0.735) | IDMN is a measure of the local homogeneity of the image transformed by wavelet filter XHH. |
| 12 | wavelet.LL_glszm_ZoneEntropy | 0.021 | 0.593  (0.514-0.672) | 0.501  (0.377-0.625) | ZE measures the uncertainty/randomness in the distribution of zone sizes and gray levels in the image transformed by wavelet filter XLL. |
| 13 | wavelet.LL_glcm_Idmn | 0.036 | 0.585  (0.506-0.664) | 0.488  (0.365-0.612) | IDMN is a measure of the local homogeneity of the image transformed by wavelet filter XLL. |
| 14 | wavelet.HH_glrlm_LongRunHighGrayLevelEmphasis | 0.030 | 0.588  (0.510-0.667) | 0.583  (0.462-0.704) | LRHGLE measures the joint distribution of long run lengths with higher gray-level values in the image transformed by wavelet filter XHH. |
| 15 | wavelet.HH_glszm_LowGrayLevelZoneEmphasis | 0.031 | 0.587  (0.509-0.666) | 0.536  (0.413-0.658) | LGLZE measures the distribution of lower gray-level size zones, with a higher value indicating a greater proportion of lower gray-level values and size zones in the image transformed by wavelet filter XHH. |
| 16 | wavelet.HH_gldm_LargeDependenceHighGrayLevelEmphasis | 0.032 | 0.587  (0.509-0.666) | 0.574  (0.453-0.696) | LDHGLE measures the joint distribution of large dependence with higher gray-level values of the image transformed by wavelet filter XHH. |
| 17 | wavelet.HL_glrlm_RunEntropy | 0.020 | 0.594  (0.516-0.673) | 0.578  (0.455-0.701) | RE measures the uncertainty/randomness in the distribution of run lengths and gray levels the image transformed by wavelet filter XHL. |
| 18 | wavelet.HH_gldm_SmallDependenceLowGrayLevelEmphasis | 0.022 | 0.593  (0.515-0.671) | 0.558  (0.436-0.680) | SDLGLE measures the joint distribution of small dependence with lower gray-level values in the image transformed by wavelet filter XHH. |
| 19 | wavelet.HH_glrlm_ShortRunLowGrayLevelEmphasis | 0.028 | 0.590  (0.511-0.668) | 0.557  (0.435-0.679) | SRLGLE measures the joint distribution of shorter run lengths with lower gray-level values in the image transformed by wavelet filter XHH. |
| 20 | wavelet.HL_glszm_GrayLevelNonUniformityNormalized | 0.036 | 0.585  (0.507-0.664) | 0.533  (0.409-0.656) | GLNN measures the similarity of gray-level intensity values in the image transformed by wavelet filter XHL. |
| **T2_tumor** | | | | | |
| **Number** | **Radiomic feature** | **p-value** | **AUC (95% CI)** | | **Description** |
|  |  |  | **Training cohort**  **(n=204)** | **Validation cohort**  **(n=88)** |  |
| 1 | wavelet.LL_glcm_Imc1 | 0.002 | 0.625  (0.547-0.704) | 0.649  (0.532-0.765) | Informational measure of correlation 1 of Gray level co-occurrence matrix of the image transformed by wavelet filter XLL. |
| 2 | wavelet.LL_firstorder_Skewness | <0.001 | 0.664  (0.589-0.739) | 0.655  (0.540-0.771) | Skewness measures the asymmetry of the distribution of values about the Mean value of the image transformed by wavelet filter XLL. |
| 3 | wavelet.HL_firstorder_Kurtosis | <0.001 | 0.643  (0.567-0.719) | 0.526  (0.403-0.650) | Kurtosis is a measure of the ‘peakedness’ of the distribution of values in the image transformed by wavelet filter XHL. |
| 4 | wavelet.LL_glszm_GrayLevelNonUniformityNormalized | <0.001 | 0.649  (0.574-0.725) | 0.561  (0.438-0.683) | GLNN measures the similarity of gray-level intensity values in the image transformed by wavelet filter XLL. |
| 5 | original_firstorder_Skewness | <0.001 | 0.665  (0.590-0.740) | 0.655  (0.539-0.770) | Skewness measures the asymmetry of the distribution of values about the Mean value of the original image. |
| 6 | wavelet.HL_firstorder_Median | <0.001 | 0.664  (0.588-0.739) | 0.567  (0.446-0.689) | The median gray level intensity within the image transformed by wavelet filter XHL. |
| 7 | original_glrlm_RunEntropy | <0.001 | 0.709  (0.639-0.780) | 0.639  (0.523-0.755) | RE measures the uncertainty/randomness in the distribution of run lengths and gray levels the original image. |
| 8 | original_glszm_GrayLevelNonUniformityNormalized | <0.001 | 0.643  (0.568-0.719) | 0.458  (0.335-0.580) | GLNN measures the similarity of gray-level intensity values in the original image. |
| 9 | wavelet.HL_gldm_LargeDependenceHighGrayLevelEmphasis | 0.033 | 0.587  (0.508-0.665) | 0.477  (0.355-0.600) | LDHGLE measures the joint distribution of large dependence with higher gray-level values of the image transformed by wavelet filter XHL. |
| 10 | wavelet.HH_firstorder_Kurtosis | 0.015 | 0.599  (0.521-0.677) | 0.524  (0.402-0.647) | Kurtosis is a measure of the ‘peakedness’ of the distribution of values in the image transformed by wavelet filter XHH. |
| 11 | original_glrlm_GrayLevelNonUniformityNormalized | <0.001 | 0.645  (0.569-0.720) | 0.574  (0.452-0.696) | GLNN measures the similarity of gray-level intensity values in the original image. |
| 12 | wavelet.LL_glrlm_LowGrayLevelRunEmphasis | 0.009 | 0.606  (0.528-0.684) | 0.486  (0.362-0.609) | LGLRE measures the distribution of low gray-level values of the image transformed by wavelet filter XLL. |
| 13 | wavelet.LL_gldm_DependenceEntropy | < 0.001 | 0.690  (0.617-0.762) | 0.620  (0.501-0.739) | Dependence entropy of Neighboring gray level dependence matrix of the image transformed by wavelet filter XLL. |
| 14 | original_ngtdm_Busyness | 0.004 | 0.617  (0.540-0.694) | 0.531  (0.408-0.653) | Busyness is a measure of the change from a pixel to its neighbor in the original image. |
| 15 | original_firstorder_Median | < 0.001 | 0.653  (0.577-0.729) | 0.500  (0.375-0.625) | The median gray level intensity within the original image. |
| 16 | wavelet.LL_firstorder_Median | < 0.001 | 0.653  (0.577-0.729) | 0.500  (0.375-0.625) | The median gray level intensity within the image transformed by wavelet filter XLL. |
| 17 | original_firstorder_Uniformity | 0.001 | 0.640  (0.564-0.716) | 0.581  (0.458-0.703) | Uniformity is a measure of the sum of the squares of each intensity value in the original image. |
| 18 | wavelet.LL_glrlm_GrayLevelNonUniformityNormalized | <0.001 | 0.643  (0.568-0.719) | 0.579  (0.457-0.701) | GLNN measures the similarity of gray-level intensity values in the image transformed by wavelet filter XLL. |
| 19 | wavelet.LL_glcm_Imc2 | <0.001 | 0.726  (0.657-0.796) | 0.662  (0.548-0.776) | Informational measure of correlation 2 of Gray level co-occurrence matrix of the image transformed by wavelet filter XLL. |
| 20 | original_glcm_Imc2 | <0.001 | 0.721  (0.652-0.791) | 0.668  (0.553-0.782) | Informational measure of correlation 2 of Gray level co-occurrence matrix of the original image. |
| **T1_edema** | | | | | |
| **Number** | **Radiomic feature** | **p-value** | **AUC (95% CI)** | | **Description** |
|  |  |  | **Training cohort**  **(n=204)** | **Validation cohort**  **(n=88)** |  |
| 1 | wavelet.LH_glcm_JointEnergy | 0.019 | 0.595  (0.517-0.673) | 0.472  (0.350-0.593) | Joint energy is a measure of homogeneous patterns in the image transformed by wavelet filter XLH. |
| 2 | wavelet.HH_glszm_LargeAreaEmphasis | < 0.001 | 0.714  (0.642-0.786) | 0.661  (0.545-0.777) | LAE is a measure of the distribution of large area size zones of the image transformed by wavelet filter XHH. |
| 3 | original_gldm_SmallDependenceLowGrayLevelEmphasis | 0.048 | 0.580  (0.501-0.659) | 0.495  (0.373-0.617) | SDLGLE measures the joint distribution of small dependence with lower gray-level values in the original image. |
| 4 | wavelet.HH_glszm_LargeAreaHighGrayLevelEmphasis | <0.001 | 0.714  (0.642-0.786) | 0.655  (0.539-0.771) | LAHGLE measures the proportion in the image of the joint distribution of larger size zones with higher gray-level values in the image transformed by wavelet filter XHH. |
| 5 | wavelet.HL_gldm_DependenceNonUniformityNormalized | 0.024 | 0.591  (0.513-0.670) | 0.644  (0.527-0.761) | DNN measures the similarity of dependence throughout the image transformed by wavelet filter XHL. |
| 6 | wavelet.HH_glszm_LargeAreaLowGrayLevelEmphasis | <0.001 | 0.710  (0.638-0.782) | 0.652  (0.536-0.768) | LALGLE measures the proportion in the image of the joint distribution of larger size zones with lower gray-level values in the image transformed by wavelet filter XHH. |
| 7 | original_glrlm_ShortRunLowGrayLevelEmphasis | 0.044 | 0.582  (0.502-0.661) | 0.503  (0.381-0.624) | SRLGLE measures the joint distribution of shorter run lengths with lower gray-level values in the original image. |
| 8 | wavelet.LL_glszm_LargeAreaHighGrayLevelEmphasis | < 0.001 | 0.671  (0.596-0.745) | 0.630  (0.514-0.747) | LAHGLE measures the proportion in the image of the joint distribution of larger size zones with higher gray-level values in the image transformed by wavelet filter XLL. |
| 9 | wavelet.LL_firstorder_TotalEnergy | <0.001 | 0.697  (0.624-0.770) | 0.611  (0.493-0.729) | Total Energy is the value of Energy feature scaled by the volume of the voxel of the image transformed by wavelet filter XLL. |
| 10 | original_firstorder_TotalEnergy | < 0.001 | 0.697  (0.624-0.770) | 0.611  (0.493-0.729) | Total Energy is the value of Energy feature scaled by the volume of the voxel of the original image. |
| 11 | wavelet.HH_glszm_ZoneVariance | <0.001 | 0.712  (0.640-0.784) | 0.659  (0.544-0.775) | ZV measures the variance in zone size volumes for the zones of the image transformed by wavelet filter XHH. |
| 12 | wavelet.LL_firstorder_Variance | < 0.001 | 0.652  (0.577-0.728) | 0.575  (0.455-0.695) | Variance is the the mean of the squared distances of each intensity value from the Mean value of the image transformed by wavelet filter XLL. |
| 13 | wavelet.LL_gldm_GrayLevelVariance | < 0.001 | 0.652  (0.577-0.728) | 0.574  (0.455-0.694) | GLV measures the variance in grey level in the image transformed by wavelet filter XLL. |
| 14 | wavelet.LL_glcm_ClusterTendency | < 0.001 | 0.653  (0.577-0.728) | 0.577  (0.457-0.696) | Cluster Tendency is a measure of groupings of voxels with similar gray-level values in the image transformed by wavelet filter XLL. |
| 15 | original_firstorder_Variance | < 0.001 | 0.652  (0.577-0.728) | 0.575  (0.455-0.695) | Variance is the the mean of the squared distances of each intensity value from the Mean value of the original image. |
| 16 | original_glcm_ClusterTendency | < 0.001 | 0.653  (0.577-0.729) | 0.575  (0.455-0.695) | Cluster Tendency is a measure of groupings of voxels with similar gray-level values in the origina image. |
| 17 | original_gldm_GrayLevelVariance | <0.001 | 0.652  (0.577-0.728) | 0.573  (0.453-0.693) | GLV measures the variance in grey level in the original image. |
| 18 | wavelet.LL_glcm_SumSquares | < 0.001 | 0.653  (0.577-0.728) | 0.577  (0.457-0.696) | Sum of Squares is a measure in the distribution of neigboring intensity level pairs about the mean intensity level in the GLCM in the image transformed by wavelet filter XLL. |
| 19 | original_glcm_SumSquares | <0.001 | 0.653  (0.577-0.729) | 0.575  (0.455-0.695) | Sum of Squares is a measure in the distribution of neigboring intensity level pairs about the mean intensity level in the GLCM in the original image. |
| 20 | wavelet.LH_glcm_Idm | 0.009 | 0.606  (0.528-0.683) | 0.497  (0.376-0.619) | IDM is a measure of the local homogeneity of the image transformed by wavelet filter XLH. |
| **CE-T1_edema** | | | | | |
| **Number** | **Radiomic feature** | **p-value** | **AUC (95% CI)** | | **Description** |
|  |  |  | **Training cohort**  **(n=204)** | **Validation cohort**  **(n=88)** |  |
| 1 | original_gldm_SmallDependenceLowGrayLevelEmphasis | 0.033 | 0.586  (0.507-0.666) | 0.501  (0.380-0.622) | SDLGLE measures the joint distribution of small dependence with lower gray-level values in the original image. |
| 2 | wavelet.LH_glcm_Idmn | < 0.001 | 0.651  (0.575-0.727) | 0.601  (0.483-0.720) | IDMN is a measure of the local homogeneity of the image transformed by wavelet filter XLH. |
| 3 | wavelet.LL_glcm_InverseVariance | 0.015 | 0.599  (0.521-0.677) | 0.548  (0.427-0.668) | ID is a measure of the local homogeneity of the image transformed by wavelet filter XLL. |
| 4 | wavelet.HH_glszm_ZoneVariance | < 0.001 | 0.710  (0.638-0.782) | 0.647  (0.531-0.763) | ZV measures the variance in zone size volumes for the zones of the image transformed by wavelet filter XHH. |
| 5 | wavelet.LH_glcm_Idm | 0.009 | 0.605  (0.527-0.683) | 0.528  (0.407-0.649) | IDM is a measure of the local homogeneity of the image transformed by wavelet filter XLH. |
| 6 | wavelet.LL_firstorder_TotalEnergy | < 0.001 | 0.706  (0.634-0.778) | 0.615  (0.497-0.733) | Total Energy is the value of Energy feature scaled by the volume of the voxel of the original image. |
| 7 | original_firstorder_TotalEnergy | < 0.001 | 0.706  (0.634-0.778) | 0.615  (0.497-0.733) | Total Energy is the value of Energy feature scaled by the volume of the voxel of the image transformed by wavelet filter XLL. |
| 8 | wavelet.LH_glcm_Id | 0.008 | 0.607  (0.634-0.779) | 0.524  (0.402-0.645) | ID is another measure of the local homogeneity of the image transformed by wavelet filter XLH. |
| 9 | wavelet.LL_glszm_LargeAreaHighGrayLevelEmphasis | < 0.001 | 0.667  (0.592-0.742) | 0.608  (0.490-0.727) | LAHGLE measures the proportion in the image of the joint distribution of larger size zones with higher gray-level values in the image transformed by wavelet filter XLL. |
| 10 | wavelet.HH_glszm_LargeAreaEmphasis | < 0.001 | 0.709  (0.637-0.781) | 0.650  (0.534-0.766) | LAE is a measure of the distribution of large area size zones of the image transformed by wavelet filter XHH. |
| 11 | original_shape_MajorAxis | 0.001 | 0.633  (0.556-0.710) | 0.623  (0.504-0.741) | Major axis length yield the largest axis length of the ROI-enclosing ellipsoid and is calculated using the largest principal component λmajor. |
| 12 | wavelet.LH_glszm_ZoneVariance | < 0.001 | 0.688  (0.615-0.761) | 0.628  (0.511-0.745) | ZV measures the variance in zone size volumes for the zones of the image transformed by wavelet filter XLH. |
| 13 | wavelet.LH_gldm_LargeDependenceEmphasis | < 0.001 | 0.643  (0.567-0.720) | 0.555  (0.435-0.676) | LDE is a measure of the distribution of large dependencies, with a greater value indicative of larger dependence and more homogeneous textures. |
| 14 | wavelet.HH_glrlm_RunVariance | < 0.001 | 0.683  (0.609-0.757) | 0.565  (0.445-0.685) | RunVariance is a measure of the variance in runs for the run lengths of the image transformed by wavelet filter XHH. |
| 15 | original_firstorder_Energy | < 0.001 | 0.704  (0.632-0.777) | 0.613  (0.495-0.731) | Energy is a measure of the magnitude of voxel values in original image. |
| 16 | wavelet.LH_glszm_LargeAreaEmphasis | < 0.001 | 0.685  (0.612-0.758) | 0.628  (0.511-0.745) | LAE is a measure of the distribution of large area size zones of the image transformed by wavelet filter XLH. |
| 17 | wavelet.LL_firstorder_Energy | < 0.001 | 0.704  (0.632-0.777) | 0.613  (0.495-0.731) | Energy is a measure of the magnitude of voxel values in the image transformed by wavelet filter XLL. |
| 18 | wavelet.HH_glcm_SumEntropy | 0.009 | 0.605  (0.528-0.683) | 0.538  (0.417-0.760) | Sum Entropy is a sum of neighborhood intensity value differences in the image transformed by wavelet filter XHH. |
| 19 | wavelet.HH_glszm_LargeAreaLowGrayLevelEmphasis | < 0.001 | 0.681  (0.607-0.755) | 0.643  (0.526-0.760) | LALGLE measures the proportion in the image of the joint distribution of larger size zones with lower gray-level values in the image transformed by wavelet filter XHH. |
| 20 | wavelet.LL_firstorder_Median | 0.009 | 0.605  (0.527-0.683) | 0.522  (0.401-0.644) | The median gray level intensity within the image transformed by wavelet filter XLL. |
| **T2_edema** | | | | | |
| **Number** | **Radiomic feature** | **p-value** | **AUC (95% CI)** | | **Description** |
|  |  |  | **Training cohort**  **(n=204)** | **Validation cohort**  **(n=88)** |  |
| 1 | wavelet.HH_glszm_LargeAreaEmphasis | < 0.001 | 0.695  (0.622-0.768) | 0.647  (0.530-0.764) | LAE is a measure of the distribution of large area size zones of the image transformed by wavelet filter XHH. |
| 2 | original_gldm_LargeDependenceHighGrayLevelEmphasis | < 0.001 | 0.691  (0.618-0.764) | 0.600  (0.481-0.719) | LDHGLE measures the joint distribution of large dependence with higher gray-level values of the original image. |
| 3 | wavelet.HH_glszm_ZoneVariance | < 0.001 | 0.696  (0.623-0.769) | 0.649  (0.532-0.765) | ZV measures the variance in zone size volumes for the zones of the image transformed by wavelet filter XHH. |
| 4 | wavelet.HL_glcm_Idm | 0.048 | 0.580  (0.502-0.658) | 0.555  (0.435-0.675) | IDM is a measure of the local homogeneity of the image transformed by wavelet filter XHL. |
| 5 | wavelet.LH_glcm_Idn | 0.001 | 0.629  (0.552-0.706) | 0.598  (0.479-0.716) | IDN is a measure of the local homogeneity of the image transformed by wavelet filter XLH. |
| 6 | wavelet.HL_glcm_Id | 0.042 | 0.582  (0.504-0.660) | 0.555  (0.435-0.675) | ID is another measure of the local homogeneity of the image transformed by wavelet filter XHL. |
| 7 | wavelet.LL_firstorder_RobustMeanAbsoluteDeviation | < 0.001 | 0.665  (0.590-0.740) | 0.598  (0.480-0.717) | Robust Mean Absolute Deviation is the mean distance of all intensity values from the Mean Value calculated on the subset of image array with gray levels in between, or equal to the 10th and 90th percentile in the image transformed by wavelet filter XLL. |
| 8 | wavelet.LH_glrlm_RunLengthNonUniformity | < 0.001 | 0.699  (0.627-0.772) | 0.635  (0.517-0.752) | RLN measures the similarity of run lengths throughout the image transformed by wavelet filter XLH. |
| 9 | wavelet.LH_glszm_SmallAreaEmphasis | 0.043 | 0.582  (0.504-0.660) | 0.524  (0.403-0.644) | SAE is a measure of the distribution of small size zones of the image transformed by wavelet filter XLH. |
| 10 | original_firstorder_RobustMeanAbsoluteDeviation | < 0.001 | 0.664  (0.589-0.740) | 0.598  (0.479-0.716) | Robust Mean Absolute Deviation is the mean distance of all intensity values from the Mean Value calculated on the subset of image array with gray levels in between, or equal to the 10th and 90th percentile in the original image. |
| 11 | original_shape_MajorAxis | 0.001 | 0.629  (0.552-0.706) | 0.625  (0.507-0.743) | Major axis length yield the largest axis length of the ROI-enclosing ellipsoid and is calculated using the largest principal component λmajor. |
| 12 | wavelet.HL_glrlm_RunLengthNonUniformity | < 0.001 | 0.692  (0.618-0.765) | 0.641  (0.524-0.758) | RLN measures the similarity of run lengths throughout the image transformed by wavelet filter XHL. |
| 13 | wavelet.LL_firstorder_InterquartileRange | < 0.001 | 0.663  (0.0.588-0.739) | 0.594  (0.476-0.713) | The interquartile range of gray values in the image transformed by wavelet filter XLL. |
| 14 | wavelet.HL_glcm_Idmn | < 0.001 | 0.669  (0.0.594-0.744) | 0.613  (0.494-0.733) | IDMN is a measure of the local homogeneity of the image transformed by wavelet filter XHL. |
| 15 | original_glrlm_LongRunHighGrayLevelEmphasis | < 0.001 | 0.689  (0.616-0.762) | 0.610  (0.492-0.729) | LRHGLE measures the joint distribution of long run lengths with higher gray-level values in the original image. |
| 16 | original_firstorder_InterquartileRange | < 0.001 | 0.662  (0.587-0.737) | 0.594  (0.475-0.712) | The interquartile range of gray values in the original image. |
| 17 | wavelet.LL_ngtdm_Busyness | 0.012 | 0.562  (0.484-0.641) | 0.609  (0.491-0.727) | Busyness is a measure of the change from a pixel to its neighbor in the image transformed by wavelet filter XLL.. |
| 18 | wavelet.LL_glcm_Imc2 | < 0.001 | 0.706  (0.633-0.779) | 0.641  (0.524-0.758) | Informational measure of correlation 2 of Gray level co-occurrence matrix of the image transformed by wavelet filter XLL. |
| 19 | wavelet.LH_glrlm_RunEntropy | 0.008 | 0.608  (0.531-0.685) | 0.572  (0.453-0.691) | RE measures the uncertainty/randomness in the distribution of run lengths and gray levels the image transformed by wavelet filter XLH. |
| 20 | wavelet.HL_glcm_Idn | < 0.001 | 0.670  (0.595-0.745) | 0.612  (0.493-0.731) | IDN is a measure of the local homogeneity of the image transformed by wavelet filter XHL. |

**Note:** AUC: area under curve; CI: confidence interval.

**Supplementary Table 4. The results of 64 modeling strategies**

| **T1_tumor** | | | | | | | | | |
| --- | --- | --- | --- | --- | --- | --- | --- | --- | --- |
| **Feature selection** | **Classifier** | **Training cohort**  **(n=204)** | | | | **Validation cohort**  **(n=88)** | | | |
|  |  | **AUC** | **ACC** | **SEN** | **SPE** | **AUC** | **ACC** | **SEN** | **SPE** |
| CFS | DT | 0.866 | 0.809 | 0.872 | 0.737 | 0.793 | 0.693 | 0.804 | 0.571 |
|  | LR | 0.662 | 0.627 | 0.468 | 0.811 | 0.789 | 0.739 | 0.652 | 0.833 |
|  | RF | 0.776 | 0.725 | 0.716 | 0.737 | 0.795 | 0.750 | 0.783 | 0.714 |
|  | SVC | 0.673 | 0.632 | 0.431 | 0.863 | 0.782 | 0.739 | 0.630 | 0.857 |
| CIFE | DT | 0.863 | 0.765 | 0.725 | 0.811 | 0.771 | 0.705 | 0.696 | 0.714 |
|  | LR | 0.680 | 0.627 | 0.450 | 0.832 | 0.783 | 0.727 | 0.630 | 0.833 |
|  | RF | 0.827 | 0.755 | 0.761 | 0.747 | 0.780 | 0.693 | 0.826 | 0.548 |
|  | SVC | 0.701 | 0.667 | 0.596 | 0.747 | 0.770 | 0.693 | 0.739 | 0.643 |
| CMIM | DT | 0.792 | 0.760 | 0.752 | 0.768 | 0.775 | 0.705 | 0.783 | 0.619 |
|  | LR | 0.691 | 0.652 | 0.541 | 0.779 | 0.781 | 0.739 | 0.739 | 0.738 |
|  | RF | 0.845 | 0.784 | 0.780 | 0.789 | 0.782 | 0.705 | 0.783 | 0.619 |
|  | SVC | 0.701 | 0.662 | 0.569 | 0.768 | 0.770 | 0.716 | 0.696 | 0.738 |
| DISR | DT | 0.815 | 0.721 | 0.642 | 0.811 | 0.778 | 0.739 | 0.783 | 0.690 |
|  | LR | 0.678 | 0.632 | 0.477 | 0.811 | 0.787 | 0.716 | 0.652 | 0.786 |
|  | RF | 0.885 | 0.814 | 0.899 | 0.716 | 0.783 | 0.693 | 0.783 | 0.595 |
|  | SVC | 0.705 | 0.672 | 0.624 | 0.726 | 0.774 | 0.693 | 0.761 | 0.619 |
| Fisher | DT | 0.817 | 0.809 | 0.954 | 0.642 | 0.791 | 0.716 | 0.870 | 0.548 |
|  | LR | 0.769 | 0.706 | 0.743 | 0.663 | 0.803 | 0.705 | 0.848 | 0.548 |
|  | RF | 0.856 | 0.779 | 0.835 | 0.716 | 0.832 | 0.761 | 0.804 | 0.714 |
|  | SVC | 0.763 | 0.706 | 0.780 | 0.621 | 0.806 | 0.705 | 0.891 | 0.500 |
| Gini | DT | 0.852 | 0.789 | 0.807 | 0.768 | 0.821 | 0.761 | 0.804 | 0.714 |
|  | LR | 0.858 | 0.784 | 0.835 | 0.726 | 0.849 | 0.761 | 0.891 | 0.619 |
|  | RF | 0.912 | 0.877 | 0.982 | 0.758 | 0.869 | 0.807 | 0.935 | 0.667 |
|  | SVC | 0.846 | 0.789 | 0.826 | 0.747 | 0.866 | 0.773 | 0.891 | 0.643 |
| ICAP | DT | 0.792 | 0.760 | 0.752 | 0.768 | 0.775 | 0.705 | 0.783 | 0.619 |
|  | LR | 0.691 | 0.652 | 0.541 | 0.779 | 0.781 | 0.739 | 0.739 | 0.738 |
|  | RF | 0.845 | 0.784 | 0.780 | 0.789 | 0.782 | 0.705 | 0.783 | 0.619 |
|  | SVC | 0.701 | 0.662 | 0.569 | 0.768 | 0.767 | 0.716 | 0.696 | 0.738 |
| JMI | DT | 0.917 | 0.882 | 1.000 | 0.747 | 0.766 | 0.693 | 0.848 | 0.524 |
|  | LR | 0.680 | 0.627 | 0.450 | 0.832 | 0.783 | 0.727 | 0.630 | 0.833 |
|  | RF | 0.839 | 0.770 | 0.688 | 0.863 | 0.787 | 0.739 | 0.717 | 0.762 |
|  | SVC | 0.702 | 0.667 | 0.596 | 0.747 | 0.770 | 0.693 | 0.739 | 0.643 |
| MCFS | DT | 0.799 | 0.765 | 1.000 | 0.495 | 0.764 | 0.648 | 0.957 | 0.310 |
|  | LR | 0.642 | 0.627 | 0.734 | 0.505 | 0.759 | 0.682 | 0.804 | 0.548 |
|  | RF | 0.770 | 0.740 | 0.817 | 0.653 | 0.773 | 0.693 | 0.870 | 0.500 |
|  | SVC | 0.730 | 0.701 | 0.661 | 0.747 | 0.780 | 0.705 | 0.717 | 0.690 |
| MIFS | DT | 0.815 | 0.740 | 0.991 | 0.453 | 0.753 | 0.625 | 0.913 | 0.310 |
|  | LR | 0.680 | 0.627 | 0.450 | 0.832 | 0.783 | 0.727 | 0.630 | 0.833 |
|  | RF | 0.950 | 0.887 | 0.881 | 0.895 | 0.783 | 0.693 | 0.717 | 0.667 |
|  | SVC | 0.702 | 0.667 | 0.596 | 0.747 | 0.770 | 0.693 | 0.739 | 0.643 |
| MIM | DT | 0.910 | 0.887 | 0.982 | 0.779 | 0.788 | 0.682 | 0.848 | 0.500 |
|  | LR | 0.690 | 0.652 | 0.505 | 0.821 | 0.787 | 0.716 | 0.652 | 0.786 |
|  | RF | 0.935 | 0.868 | 0.807 | 0.937 | 0.777 | 0.705 | 0.696 | 0.714 |
|  | SVC | 0.700 | 0.662 | 0.541 | 0.800 | 0.771 | 0.727 | 0.674 | 0.786 |
| MRMR | DT | 0.815 | 0.740 | 0.991 | 0.453 | 0.753 | 0.625 | 0.913 | 0.310 |
|  | LR | 0.680 | 0.627 | 0.450 | 0.832 | 0.783 | 0.727 | 0.630 | 0.833 |
|  | RF | 0.950 | 0.887 | 0.881 | 0.895 | 0.783 | 0.693 | 0.717 | 0.667 |
|  | SVC | 0.702 | 0.667 | 0.596 | 0.747 | 0.770 | 0.693 | 0.739 | 0.643 |
| Relief | DT | 0.856 | 0.804 | 0.991 | 0.589 | 0.823 | 0.727 | 0.935 | 0.500 |
|  | LR | 0.737 | 0.706 | 0.835 | 0.558 | 0.831 | 0.750 | 0.848 | 0.643 |
|  | RF | 0.827 | 0.775 | 0.872 | 0.663 | 0.857 | 0.739 | 0.870 | 0.595 |
|  | SVC | 0.765 | 0.711 | 0.807 | 0.600 | 0.862 | 0.773 | 0.935 | 0.595 |
| RFS | DT | 0.859 | 0.819 | 0.972 | 0.642 | 0.818 | 0.716 | 0.891 | 0.524 |
|  | LR | 0.778 | 0.725 | 0.844 | 0.589 | 0.808 | 0.727 | 0.913 | 0.524 |
|  | RF | 0.956 | 0.892 | 0.872 | 0.916 | 0.840 | 0.795 | 0.783 | 0.810 |
|  | SVC | 0.789 | 0.740 | 0.835 | 0.632 | 0.803 | 0.705 | 0.848 | 0.548 |
| SPEC | DT | 0.768 | 0.721 | 0.872 | 0.547 | 0.822 | 0.773 | 0.891 | 0.643 |
|  | LR | 0.762 | 0.711 | 0.752 | 0.663 | 0.802 | 0.716 | 0.870 | 0.548 |
|  | RF | 0.898 | 0.848 | 0.917 | 0.768 | 0.853 | 0.750 | 0.804 | 0.690 |
|  | SVC | 0.735 | 0.686 | 0.615 | 0.768 | 0.833 | 0.716 | 0.739 | 0.690 |
| Trace ratio | DT | 0.845 | 0.824 | 0.954 | 0.674 | 0.711 | 0.682 | 0.804 | 0.548 |
|  | LR | 0.637 | 0.618 | 0.495 | 0.758 | 0.762 | 0.727 | 0.674 | 0.786 |
|  | RF | 0.731 | 0.706 | 0.853 | 0.537 | 0.758 | 0.648 | 0.891 | 0.381 |
|  | SVC | 0.633 | 0.603 | 0.431 | 0.800 | 0.747 | 0.670 | 0.630 | 0.714 |
| **CE-T1_tumor** | | | | | | | | | |
| **Feature selection** | **Classifier** | **Training cohort**  **(n=204)** | | | | **Validation cohort**  **(n=88)** | | | |
|  |  | **AUC** | **ACC** | **SEN** | **SPE** | **AUC** | **ACC** | **SEN** | **SPE** |
| CFS | DT | 0.818 | 0.760 | 0.936 | 0.558 | 0.647 | 0.602 | 0.848 | 0.333 |
|  | LR | 0.682 | 0.657 | 0.688 | 0.621 | 0.551 | 0.489 | 0.565 | 0.405 |
|  | RF | 0.934 | 0.887 | 0.881 | 0.895 | 0.612 | 0.568 | 0.609 | 0.524 |
|  | SVC | 1.000 | 1.000 | 1.000 | 1.000 | 0.693 | 0.591 | 0.370 | 0.833 |
| CIFE | DT | 0.918 | 0.887 | 0.963 | 0.800 | 0.713 | 0.636 | 0.804 | 0.452 |
|  | LR | 0.741 | 0.711 | 0.789 | 0.621 | 0.602 | 0.511 | 0.630 | 0.381 |
|  | RF | 0.883 | 0.804 | 0.798 | 0.811 | 0.651 | 0.602 | 0.609 | 0.595 |
|  | SVC | 0.868 | 0.843 | 0.853 | 0.832 | 0.720 | 0.693 | 0.630 | 0.762 |
| CMIM | DT | 0.901 | 0.858 | 1.000 | 0.695 | 0.763 | 0.716 | 0.891 | 0.524 |
|  | LR | 0.741 | 0.711 | 0.789 | 0.621 | 0.602 | 0.511 | 0.630 | 0.381 |
|  | RF | 0.752 | 0.750 | 0.890 | 0.589 | 0.690 | 0.670 | 0.870 | 0.452 |
|  | SVC | 0.868 | 0.843 | 0.853 | 0.832 | 0.720 | 0.693 | 0.630 | 0.762 |
| DISR | DT | 0.837 | 0.745 | 0.661 | 0.842 | 0.733 | 0.670 | 0.630 | 0.714 |
|  | LR | 0.742 | 0.706 | 0.761 | 0.642 | 0.601 | 0.534 | 0.609 | 0.452 |
|  | RF | 0.719 | 0.701 | 0.817 | 0.568 | 0.693 | 0.648 | 0.804 | 0.476 |
|  | SVC | 0.868 | 0.843 | 0.853 | 0.832 | 0.720 | 0.693 | 0.630 | 0.762 |
| Fisher | DT | 0.905 | 0.887 | 1.000 | 0.758 | 0.727 | 0.625 | 0.826 | 0.405 |
|  | LR | 0.713 | 0.696 | 0.972 | 0.379 | 0.659 | 0.557 | 0.848 | 0.238 |
|  | RF | 0.908 | 0.838 | 0.872 | 0.800 | 0.685 | 0.670 | 0.717 | 0.619 |
|  | SVC | 0.874 | 0.833 | 0.881 | 0.779 | 0.758 | 0.705 | 0.696 | 0.714 |
| Gini | DT | 0.960 | 0.907 | 0.991 | 0.811 | 0.731 | 0.739 | 0.848 | 0.619 |
|  | LR | 0.714 | 0.696 | 0.752 | 0.632 | 0.619 | 0.580 | 0.674 | 0.476 |
|  | RF | 0.897 | 0.833 | 0.927 | 0.726 | 0.692 | 0.648 | 0.783 | 0.500 |
|  | SVC | 0.822 | 0.819 | 0.954 | 0.663 | 0.753 | 0.682 | 0.804 | 0.548 |
| ICAP | DT | 0.901 | 0.858 | 1.000 | 0.695 | 0.763 | 0.716 | 0.891 | 0.524 |
|  | LR | 0.741 | 0.711 | 0.789 | 0.621 | 0.602 | 0.511 | 0.630 | 0.381 |
|  | RF | 0.752 | 0.750 | 0.890 | 0.589 | 0.690 | 0.670 | 0.870 | 0.452 |
|  | SVC | 0.868 | 0.843 | 0.853 | 0.832 | 0.720 | 0.693 | 0.630 | 0.762 |
| JMI | DT | 0.918 | 0.887 | 0.963 | 0.800 | 0.713 | 0.636 | 0.804 | 0.452 |
|  | LR | 0.741 | 0.711 | 0.789 | 0.621 | 0.602 | 0.511 | 0.630 | 0.381 |
|  | RF | 0.883 | 0.804 | 0.798 | 0.811 | 0.651 | 0.602 | 0.609 | 0.595 |
|  | SVC | 0.868 | 0.843 | 0.853 | 0.832 | 0.720 | 0.693 | 0.630 | 0.762 |
| MCFS | DT | 0.950 | 0.907 | 0.954 | 0.853 | 0.721 | 0.614 | 0.630 | 0.595 |
|  | LR | 0.706 | 0.657 | 0.679 | 0.632 | 0.629 | 0.580 | 0.565 | 0.595 |
|  | RF | 0.939 | 0.892 | 0.899 | 0.884 | 0.665 | 0.580 | 0.609 | 0.548 |
|  | SVC | 0.838 | 0.819 | 0.936 | 0.684 | 0.753 | 0.670 | 0.783 | 0.548 |
| MIFS | DT | 0.918 | 0.887 | 0.963 | 0.800 | 0.713 | 0.636 | 0.804 | 0.452 |
|  | LR | 0.741 | 0.711 | 0.789 | 0.621 | 0.602 | 0.511 | 0.630 | 0.381 |
|  | RF | 0.883 | 0.804 | 0.798 | 0.811 | 0.651 | 0.602 | 0.609 | 0.595 |
|  | SVC | 0.868 | 0.843 | 0.853 | 0.832 | 0.721 | 0.693 | 0.630 | 0.762 |
| MIM | DT | 0.837 | 0.745 | 0.661 | 0.842 | 0.733 | 0.670 | 0.630 | 0.714 |
|  | LR | 0.742 | 0.706 | 0.761 | 0.642 | 0.601 | 0.534 | 0.609 | 0.452 |
|  | RF | 0.719 | 0.701 | 0.817 | 0.568 | 0.693 | 0.648 | 0.804 | 0.476 |
|  | SVC | 0.864 | 0.858 | 0.881 | 0.832 | 0.719 | 0.682 | 0.609 | 0.762 |
| MRMR | DT | 0.918 | 0.887 | 0.963 | 0.800 | 0.713 | 0.636 | 0.804 | 0.452 |
|  | LR | 0.741 | 0.711 | 0.789 | 0.621 | 0.602 | 0.511 | 0.630 | 0.381 |
|  | RF | 0.883 | 0.804 | 0.798 | 0.811 | 0.651 | 0.602 | 0.609 | 0.595 |
|  | SVC | 0.868 | 0.843 | 0.853 | 0.832 | 0.720 | 0.693 | 0.630 | 0.762 |
| Relief | DT | 0.737 | 0.672 | 0.853 | 0.463 | 0.700 | 0.693 | 0.935 | 0.429 |
|  | LR | 0.721 | 0.691 | 0.716 | 0.663 | 0.607 | 0.580 | 0.565 | 0.595 |
|  | RF | 0.956 | 0.897 | 0.881 | 0.916 | 0.613 | 0.591 | 0.587 | 0.595 |
|  | SVC | 0.863 | 0.868 | 0.899 | 0.832 | 0.695 | 0.648 | 0.565 | 0.738 |
| RFS | DT | 0.811 | 0.755 | 0.725 | 0.789 | 0.731 | 0.648 | 0.730 | 0.667 |
|  | LR | 0.713 | 0.676 | 0.761 | 0.579 | 0.629 | 0.545 | 0.652 | 0.429 |
|  | RF | 0.827 | 0.770 | 0.835 | 0.695 | 0.666 | 0.670 | 0.717 | 0.619 |
|  | SVC | 0.878 | 0.838 | 0.844 | 0.832 | 0.724 | 0.682 | 0.630 | 0.738 |
| SPEC | DT | 0.923 | 0.873 | 0.963 | 0.768 | 0.719 | 0.625 | 0.761 | 0.476 |
|  | LR | 0.745 | 0.725 | 0.780 | 0.663 | 0.644 | 0.580 | 0.652 | 0.500 |
|  | RF | 0.800 | 0.735 | 0.817 | 0.642 | 0.679 | 0.636 | 0.783 | 0.476 |
|  | SVC | 0.872 | 0.833 | 0.927 | 0.726 | 0.737 | 0.693 | 0.783 | 0.595 |
| Trace ratio | DT | 0.824 | 0.784 | 0.927 | 0.621 | 0.674 | 0.625 | 0.826 | 0.405 |
|  | LR | 0.741 | 0.701 | 0.862 | 0.516 | 0.577 | 0.523 | 0.696 | 0.333 |
|  | RF | 0.971 | 0.922 | 0.917 | 0.926 | 0.622 | 0.557 | 0.630 | 0.476 |
|  | SVC | 0.863 | 0.843 | 0.872 | 0.811 | 0.675 | 0.648 | 0.565 | 0.738 |
| **T2_tumor** | | | | | | | | | |
| **Feature selection** | **Classifier** | **Training cohort**  **(n=204)** | | | | **Validation cohort**  **(n=88)** | | | |
|  |  | **AUC** | **ACC** | **SEN** | **SPE** | **AUC** | **ACC** | **SEN** | **SPE** |
| CFS | DT | 0.889 | 0.833 | 0.908 | 0.747 | 0.732 | 0.659 | 0.783 | 0.524 |
|  | LR | 0.726 | 0.711 | 0.771 | 0.642 | 0.672 | 0.670 | 0.761 | 0.571 |
|  | RF | 0.941 | 0.868 | 0.927 | 0.800 | 0.696 | 0.705 | 0.696 | 0.714 |
|  | SVC | 0.674 | 0.667 | 0.651 | 0.684 | 0.730 | 0.693 | 0.739 | 0.643 |
| CIFE | DT | 0.921 | 0.877 | 0.991 | 0.747 | 0.714 | 0.648 | 0.717 | 0.571 |
|  | LR | 0.720 | 0.711 | 0.771 | 0.642 | 0.672 | 0.693 | 0.783 | 0.595 |
|  | RF | 0.853 | 0.799 | 0.817 | 0.779 | 0.664 | 0.591 | 0.609 | 0.571 |
|  | SVC | 0.669 | 0.657 | 0.642 | 0.674 | 0.716 | 0.670 | 0.674 | 0.667 |
| CMIM | DT | 0.949 | 0.922 | 1.000 | 0.832 | 0.708 | 0.648 | 0.739 | 0.548 |
|  | LR | 0.755 | 0.730 | 0.798 | 0.653 | 0.725 | 0.636 | 0.783 | 0.476 |
|  | RF | 0.813 | 0.755 | 0.780 | 0.726 | 0.680 | 0.602 | 0.630 | 0.571 |
|  | SVC | 0.763 | 0.725 | 0.761 | 0.684 | 0.761 | 0.682 | 0.761 | 0.595 |
| DISR | DT | 0.964 | 0.922 | 0.963 | 0.874 | 0.738 | 0.693 | 0.674 | 0.714 |
|  | LR | 0.731 | 0.711 | 0.734 | 0.684 | 0.669 | 0.659 | 0.717 | 0.595 |
|  | RF | 0.875 | 0.828 | 0.743 | 0.926 | 0.659 | 0.580 | 0.435 | 0.738 |
|  | SVC | 0.672 | 0.642 | 0.633 | 0.653 | 0.726 | 0.682 | 0.674 | 0.690 |
| Fisher | DT | 0.871 | 0.809 | 0.872 | 0.737 | 0.726 | 0.682 | 0.848 | 0.500 |
|  | LR | 0.754 | 0.706 | 0.761 | 0.642 | 0.719 | 0.682 | 0.761 | 0.595 |
|  | RF | 0.863 | 0.804 | 0.789 | 0.821 | 0.706 | 0.659 | 0.739 | 0.571 |
|  | SVC | 0.801 | 0.770 | 0.743 | 0.800 | 0.733 | 0.670 | 0.609 | 0.738 |
| Gini | DT | 0.889 | 0.819 | 1.000 | 0.611 | 0.728 | 0.693 | 0.870 | 0.500 |
|  | LR | 0.781 | 0.725 | 0.679 | 0.779 | 0.722 | 0.670 | 0.652 | 0.690 |
|  | RF | 0.917 | 0.853 | 0.789 | 0.926 | 0.700 | 0.670 | 0.630 | 0.714 |
|  | SVC | 0.808 | 0.775 | 0.752 | 0.800 | 0.730 | 0.659 | 0.587 | 0.738 |
| ICAP | DT | 0.949 | 0.922 | 1.000 | 0.832 | 0.708 | 0.648 | 0.739 | 0.548 |
|  | LR | 0.755 | 0.730 | 0.798 | 0.653 | 0.725 | 0.636 | 0.783 | 0.476 |
|  | RF | 0.813 | 0.755 | 0.780 | 0.726 | 0.680 | 0.614 | 0.696 | 0.524 |
|  | SVC | 0.763 | 0.725 | 0.761 | 0.684 | 0.760 | 0.682 | 0.761 | 0.595 |
| JMI | DT | 0.908 | 0.853 | 0.972 | 0.716 | 0.733 | 0.659 | 0.804 | 0.500 |
|  | LR | 0.722 | 0.716 | 0.780 | 0.642 | 0.670 | 0.682 | 0.783 | 0.571 |
|  | RF | 0.954 | 0.882 | 0.853 | 0.916 | 0.662 | 0.648 | 0.565 | 0.738 |
|  | SVC | 0.663 | 0.652 | 0.706 | 0.589 | 0.715 | 0.670 | 0.739 | 0.595 |
| MCFS | DT | 0.772 | 0.745 | 0.862 | 0.611 | 0.704 | 0.659 | 0.739 | 0.571 |
|  | LR | 0.818 | 0.755 | 0.798 | 0.705 | 0.703 | 0.636 | 0.783 | 0.476 |
|  | RF | 0.888 | 0.828 | 0.761 | 0.905 | 0.655 | 0.659 | 0.674 | 0.643 |
|  | SVC | 0.856 | 0.799 | 0.917 | 0.663 | 0.714 | 0.648 | 0.848 | 0.429 |
| MIFS | DT | 0.885 | 0.838 | 1.000 | 0.653 | 0.751 | 0.659 | 0.913 | 0.381 |
|  | LR | 0.720 | 0.711 | 0.771 | 0.642 | 0.672 | 0.693 | 0.783 | 0.595 |
|  | RF | 0.913 | 0.833 | 0.945 | 0.705 | 0.635 | 0.580 | 0.717 | 0.429 |
|  | SVC | 0.669 | 0.657 | 0.642 | 0.674 | 0.716 | 0.670 | 0.674 | 0.667 |
| MIM | DT | 0.924 | 0.882 | 0.972 | 0.779 | 0.742 | 0.659 | 0.739 | 0.571 |
|  | LR | 0.755 | 0.721 | 0.771 | 0.663 | 0.725 | 0.636 | 0.783 | 0.476 |
|  | RF | 0.821 | 0.750 | 0.743 | 0.758 | 0.665 | 0.614 | 0.587 | 0.643 |
|  | SVC | 0.735 | 0.696 | 0.670 | 0.726 | 0.743 | 0.670 | 0.739 | 0.595 |
| MRMR | DT | 0.804 | 0.750 | 0.752 | 0.747 | 0.741 | 0.693 | 0.674 | 0.714 |
|  | LR | 0.720 | 0.711 | 0.771 | 0.642 | 0.672 | 0.693 | 0.783 | 0.595 |
|  | RF | 0.886 | 0.828 | 0.826 | 0.832 | 0.646 | 0.636 | 0.609 | 0.667 |
|  | SVC | 0.669 | 0.657 | 0.642 | 0.674 | 0.716 | 0.670 | 0.674 | 0.667 |
| Relief | DT | 0.926 | 0.917 | 1.000 | 0.821 | 0.685 | 0.648 | 0.717 | 0.571 |
|  | LR | 0.739 | 0.691 | 0.826 | 0.537 | 0.670 | 0.591 | 0.826 | 0.333 |
|  | RF | 0.883 | 0.819 | 0.835 | 0.800 | 0.688 | 0.659 | 0.804 | 0.500 |
|  | SVC | 0.738 | 0.676 | 0.569 | 0.800 | 0.680 | 0.625 | 0.543 | 0.714 |
| RFS | DT | 0.858 | 0.794 | 0.927 | 0.642 | 0.762 | 0.693 | 0.848 | 0.524 |
|  | LR | 0.785 | 0.740 | 0.817 | 0.653 | 0.711 | 0.614 | 0.783 | 0.429 |
|  | RF | 0.832 | 0.755 | 0.688 | 0.832 | 0.695 | 0.670 | 0.630 | 0.714 |
|  | SVC | 0.783 | 0.740 | 0.835 | 0.632 | 0.702 | 0.648 | 0.826 | 0.452 |
| SPEC | DT | 0.953 | 0.902 | 0.972 | 0.821 | 0.720 | 0.602 | 0.761 | 0.429 |
|  | LR | 0.767 | 0.711 | 0.789 | 0.621 | 0.706 | 0.636 | 0.783 | 0.476 |
|  | RF | 0.873 | 0.824 | 0.862 | 0.779 | 0.744 | 0.693 | 0.783 | 0.595 |
|  | SVC | 0.807 | 0.755 | 0.725 | 0.789 | 0.718 | 0.705 | 0.717 | 0.690 |
| Trace ratio | DT | 0.907 | 0.814 | 0.752 | 0.884 | 0.632 | 0.625 | 0.587 | 0.667 |
|  | LR | 0.757 | 0.730 | 0.862 | 0.579 | 0.690 | 0.636 | 0.826 | 0.429 |
|  | RF | 0.786 | 0.755 | 0.853 | 0.642 | 0.620 | 0.568 | 0.739 | 0.381 |
|  | SVC | 0.668 | 0.686 | 0.734 | 0.632 | 0.726 | 0.659 | 0.739 | 0.571 |
| **T1_edema** | | | | | | | | | |
| **Feature selection** | **Classifier** | **Training cohort**  **(n=204)** | | | | **Validation cohort**  **(n=88)** | | | |
|  |  | **AUC** | **ACC** | **SEN** | **SPE** | **AUC** | **ACC** | **SEN** | **SPE** |
| CFS | DT | 0.960 | 0.907 | 1.000 | 0.800 | 0.701 | 0.659 | 0.804 | 0.500 |
|  | LR | 0.682 | 0.652 | 0.670 | 0.632 | 0.673 | 0.670 | 0.761 | 0.571 |
|  | RF | 0.700 | 0.662 | 0.560 | 0.779 | 0.683 | 0.625 | 0.630 | 0.619 |
|  | SVC | 0.660 | 0.647 | 0.679 | 0.611 | 0.707 | 0.602 | 0.696 | 0.500 |
| CIFE | DT | 0.831 | 0.819 | 0.991 | 0.621 | 0.745 | 0.670 | 0.913 | 0.405 |
|  | LR | 0.723 | 0.696 | 0.725 | 0.663 | 0.652 | 0.591 | 0.717 | 0.452 |
|  | RF | 0.726 | 0.672 | 0.752 | 0.579 | 0.663 | 0.636 | 0.739 | 0.524 |
|  | SVC | 0.729 | 0.676 | 0.651 | 0.705 | 0.677 | 0.614 | 0.674 | 0.548 |
| CMIM | DT | 0.811 | 0.799 | 1.000 | 0.568 | 0.702 | 0.625 | 0.913 | 0.310 |
|  | LR | 0.722 | 0.676 | 0.633 | 0.726 | 0.674 | 0.636 | 0.674 | 0.595 |
|  | RF | 0.735 | 0.725 | 0.826 | 0.611 | 0.675 | 0.625 | 0.761 | 0.476 |
|  | SVC | 0.778 | 0.775 | 0.872 | 0.663 | 0.703 | 0.659 | 0.761 | 0.548 |
| DISR | DT | 0.812 | 0.789 | 1.000 | 0.547 | 0.718 | 0.614 | 1.000 | 0.190 |
|  | LR | 0.722 | 0.681 | 0.679 | 0.684 | 0.674 | 0.614 | 0.674 | 0.548 |
|  | RF | 0.787 | 0.765 | 0.908 | 0.600 | 0.690 | 0.648 | 0.826 | 0.452 |
|  | SVC | 0.778 | 0.775 | 0.872 | 0.663 | 0.703 | 0.659 | 0.761 | 0.548 |
| Fisher | DT | 0.811 | 0.779 | 0.908 | 0.632 | 0.721 | 0.670 | 0.935 | 0.381 |
|  | LR | 0.728 | 0.725 | 0.881 | 0.547 | 0.698 | 0.625 | 0.848 | 0.381 |
|  | RF | 0.877 | 0.833 | 0.862 | 0.800 | 0.695 | 0.625 | 0.696 | 0.548 |
|  | SVC | 0.706 | 0.735 | 0.908 | 0.537 | 0.753 | 0.682 | 0.848 | 0.500 |
| Gini | DT | 0.854 | 0.789 | 0.798 | 0.779 | 0.709 | 0.670 | 0.783 | 0.548 |
|  | LR | 0.728 | 0.667 | 0.569 | 0.779 | 0.689 | 0.614 | 0.587 | 0.643 |
|  | RF | 0.887 | 0.848 | 0.899 | 0.789 | 0.690 | 0.614 | 0.783 | 0.429 |
|  | SVC | 0.704 | 0.676 | 0.890 | 0.432 | 0.702 | 0.636 | 0.935 | 0.310 |
| ICAP | DT | 0.811 | 0.799 | 1.000 | 0.568 | 0.702 | 0.625 | 0.913 | 0.310 |
|  | LR | 0.722 | 0.676 | 0.633 | 0.726 | 0.674 | 0.636 | 0.674 | 0.595 |
|  | RF | 0.735 | 0.725 | 0.826 | 0.611 | 0.675 | 0.625 | 0.761 | 0.476 |
|  | SVC | 0.778 | 0.775 | 0.872 | 0.663 | 0.703 | 0.659 | 0.761 | 0.548 |
| JMI | DT | 0.846 | 0.824 | 0.991 | 0.632 | 0.734 | 0.648 | 0.935 | 0.333 |
|  | LR | 0.737 | 0.725 | 0.807 | 0.632 | 0.659 | 0.591 | 0.804 | 0.357 |
|  | RF | 0.858 | 0.833 | 0.972 | 0.674 | 0.660 | 0.580 | 0.804 | 0.333 |
|  | SVC | 0.718 | 0.691 | 0.752 | 0.621 | 0.699 | 0.614 | 0.761 | 0.452 |
| MCFS | DT | 0.961 | 0.907 | 1.000 | 0.800 | 0.709 | 0.693 | 0.913 | 0.452 |
|  | LR | 0.745 | 0.691 | 0.624 | 0.768 | 0.690 | 0.636 | 0.652 | 0.619 |
|  | RF | 0.890 | 0.853 | 0.945 | 0.747 | 0.692 | 0.614 | 0.804 | 0.405 |
|  | SVC | 0.729 | 0.686 | 0.798 | 0.558 | 0.694 | 0.614 | 0.761 | 0.452 |
| MIFS | DT | 0.840 | 0.824 | 1.000 | 0.621 | 0.750 | 0.648 | 0.978 | 0.286 |
|  | LR | 0.736 | 0.711 | 0.780 | 0.632 | 0.662 | 0.591 | 0.783 | 0.381 |
|  | RF | 0.723 | 0.701 | 0.844 | 0.537 | 0.682 | 0.659 | 0.913 | 0.381 |
|  | SVC | 0.732 | 0.711 | 0.761 | 0.653 | 0.687 | 0.591 | 0.739 | 0.429 |
| MIM | DT | 0.970 | 0.922 | 1.000 | 0.832 | 0.708 | 0.693 | 0.848 | 0.524 |
|  | LR | 0.754 | 0.696 | 0.807 | 0.568 | 0.688 | 0.625 | 0.804 | 0.429 |
|  | RF | 0.810 | 0.814 | 0.954 | 0.653 | 0.667 | 0.659 | 0.848 | 0.452 |
|  | SVC | 0.733 | 0.725 | 0.853 | 0.579 | 0.709 | 0.659 | 0.826 | 0.476 |
| MRMR | DT | 0.836 | 0.819 | 0.991 | 0.621 | 0.741 | 0.659 | 0.913 | 0.381 |
|  | LR | 0.758 | 0.730 | 0.807 | 0.642 | 0.651 | 0.636 | 0.761 | 0.500 |
|  | RF | 0.761 | 0.711 | 0.853 | 0.547 | 0.688 | 0.602 | 0.848 | 0.333 |
|  | SVC | 0.716 | 0.706 | 0.798 | 0.600 | 0.690 | 0.636 | 0.804 | 0.452 |
| Relief | DT | 0.831 | 0.799 | 0.972 | 0.600 | 0.717 | 0.625 | 1.000 | 0.214 |
|  | LR | 0.773 | 0.750 | 0.890 | 0.589 | 0.734 | 0.648 | 0.848 | 0.429 |
|  | RF | 0.818 | 0.799 | 0.927 | 0.653 | 0.731 | 0.705 | 0.913 | 0.476 |
|  | SVC | 0.691 | 0.706 | 0.835 | 0.558 | 0.753 | 0.693 | 0.848 | 0.524 |
| RFS | DT | 0.768 | 0.716 | 0.927 | 0.474 | 0.673 | 0.648 | 0.957 | 0.310 |
|  | LR | 0.766 | 0.721 | 0.807 | 0.621 | 0.728 | 0.659 | 0.848 | 0.452 |
|  | RF | 0.944 | 0.897 | 0.936 | 0.853 | 0.692 | 0.614 | 0.804 | 0.405 |
|  | SVC | 0.834 | 0.814 | 0.890 | 0.726 | 0.730 | 0.682 | 0.717 | 0.643 |
| SPEC | DT | 0.904 | 0.868 | 1.000 | 0.716 | 0.729 | 0.705 | 0.935 | 0.452 |
|  | LR | 0.778 | 0.750 | 0.780 | 0.716 | 0.747 | 0.670 | 0.848 | 0.476 |
|  | RF | 0.918 | 0.833 | 0.862 | 0.800 | 0.718 | 0.625 | 0.804 | 0.429 |
|  | SVC | 0.799 | 0.755 | 0.817 | 0.684 | 0.784 | 0.693 | 0.848 | 0.524 |
| Trace ratio | DT | 0.860 | 0.838 | 1.000 | 0.653 | 0.726 | 0.648 | 0.913 | 0.357 |
|  | LR | 0.799 | 0.716 | 0.624 | 0.821 | 0.717 | 0.670 | 0.739 | 0.595 |
|  | RF | 0.916 | 0.897 | 0.972 | 0.811 | 0.705 | 0.648 | 0.739 | 0.548 |
|  | SVC | 0.853 | 0.789 | 0.853 | 0.716 | 0.756 | 0.670 | 0.826 | 0.500 |
| **CE-T1_edema** | | | | | | | | | |
| **Feature selection** | **Classifier** | **Training cohort**  **(n=204)** | | | | **Validation cohort**  **(n=88)** | | | |
|  |  | **AUC** | **ACC** | **SEN** | **SPE** | **AUC** | **ACC** | **SEN** | **SPE** |
| CFS | DT | 0.896 | 0.848 | 1.000 | 0.674 | 0.697 | 0.648 | 0.848 | 0.429 |
|  | LR | 0.715 | 0.676 | 0.817 | 0.516 | 0.662 | 0.568 | 0.783 | 0.333 |
|  | RF | 0.929 | 0.887 | 0.945 | 0.821 | 0.659 | 0.580 | 0.696 | 0.452 |
|  | SVC | 0.683 | 0.701 | 0.807 | 0.579 | 0.745 | 0.716 | 0.891 | 0.524 |
| CIFE | DT | 0.914 | 0.863 | 0.963 | 0.747 | 0.732 | 0.693 | 0.826 | 0.548 |
|  | LR | 0.732 | 0.681 | 0.661 | 0.705 | 0.690 | 0.625 | 0.652 | 0.595 |
|  | RF | 0.807 | 0.765 | 0.927 | 0.579 | 0.714 | 0.648 | 0.848 | 0.429 |
|  | SVC | 0.714 | 0.676 | 0.798 | 0.537 | 0.692 | 0.614 | 0.783 | 0.429 |
| CMIM | DT | 0.917 | 0.863 | 0.972 | 0.737 | 0.746 | 0.705 | 0.891 | 0.500 |
|  | LR | 0.742 | 0.681 | 0.615 | 0.758 | 0.695 | 0.602 | 0.609 | 0.595 |
|  | RF | 0.926 | 0.873 | 0.917 | 0.821 | 0.695 | 0.636 | 0.696 | 0.571 |
|  | SVC | 0.688 | 0.681 | 0.844 | 0.495 | 0.697 | 0.659 | 0.891 | 0.405 |
| DISR | DT | 0.971 | 0.922 | 0.991 | 0.842 | 0.723 | 0.670 | 0.826 | 0.500 |
|  | LR | 0.750 | 0.706 | 0.706 | 0.705 | 0.699 | 0.602 | 0.674 | 0.524 |
|  | RF | 0.931 | 0.877 | 0.899 | 0.853 | 0.726 | 0.659 | 0.739 | 0.571 |
|  | SVC | 0.782 | 0.755 | 0.780 | 0.726 | 0.698 | 0.636 | 0.717 | 0.548 |
| Fisher | DT | 0.917 | 0.882 | 0.991 | 0.758 | 0.707 | 0.682 | 0.891 | 0.452 |
|  | LR | 0.700 | 0.662 | 0.651 | 0.674 | 0.672 | 0.636 | 0.674 | 0.595 |
|  | RF | 0.801 | 0.779 | 0.899 | 0.642 | 0.697 | 0.693 | 0.891 | 0.476 |
|  | SVC | 0.712 | 0.667 | 0.670 | 0.663 | 0.712 | 0.648 | 0.696 | 0.595 |
| Gini | DT | 0.913 | 0.868 | 0.982 | 0.737 | 0.718 | 0.727 | 0.891 | 0.548 |
|  | LR | 0.741 | 0.706 | 0.780 | 0.621 | 0.697 | 0.625 | 0.783 | 0.452 |
|  | RF | 0.777 | 0.740 | 0.807 | 0.663 | 0.681 | 0.602 | 0.783 | 0.405 |
|  | SVC | 0.742 | 0.706 | 0.936 | 0.442 | 0.695 | 0.648 | 0.978 | 0.286 |
| ICAP | DT | 0.917 | 0.863 | 0.972 | 0.737 | 0.746 | 0.705 | 0.891 | 0.500 |
|  | LR | 0.742 | 0.681 | 0.615 | 0.758 | 0.695 | 0.602 | 0.609 | 0.595 |
|  | RF | 0.926 | 0.873 | 0.917 | 0.821 | 0.695 | 0.636 | 0.696 | 0.571 |
|  | SVC | 0.689 | 0.686 | 0.872 | 0.474 | 0.695 | 0.659 | 0.891 | 0.405 |
| JMI | DT | 0.885 | 0.843 | 1.000 | 0.663 | 0.724 | 0.670 | 0.848 | 0.476 |
|  | LR | 0.752 | 0.696 | 0.670 | 0.726 | 0.689 | 0.591 | 0.609 | 0.571 |
|  | RF | 0.753 | 0.696 | 0.670 | 0.726 | 0.708 | 0.648 | 0.717 | 0.571 |
|  | SVC | 0.742 | 0.681 | 0.624 | 0.747 | 0.692 | 0.591 | 0.587 | 0.595 |
| MCFS | DT | 0.935 | 0.882 | 1.000 | 0.747 | 0.730 | 0.693 | 0.891 | 0.476 |
|  | LR | 0.739 | 0.686 | 0.807 | 0.547 | 0.687 | 0.614 | 0.826 | 0.381 |
|  | RF | 0.853 | 0.838 | 0.963 | 0.695 | 0.699 | 0.591 | 0.761 | 0.405 |
|  | SVC | 0.737 | 0.681 | 0.651 | 0.716 | 0.682 | 0.591 | 0.630 | 0.548 |
| MIFS | DT | 0.924 | 0.868 | 0.954 | 0.768 | 0.692 | 0.659 | 0.826 | 0.476 |
|  | LR | 0.731 | 0.696 | 0.862 | 0.505 | 0.689 | 0.636 | 0.870 | 0.381 |
|  | RF | 0.846 | 0.804 | 0.908 | 0.684 | 0.687 | 0.602 | 0.848 | 0.333 |
|  | SVC | 0.711 | 0.681 | 0.807 | 0.537 | 0.693 | 0.602 | 0.783 | 0.405 |
| MIM | DT | 0.925 | 0.873 | 1.000 | 0.726 | 0.701 | 0.705 | 0.913 | 0.476 |
|  | LR | 0.770 | 0.740 | 0.899 | 0.558 | 0.732 | 0.670 | 0.913 | 0.405 |
|  | RF | 0.895 | 0.838 | 0.890 | 0.779 | 0.721 | 0.670 | 0.717 | 0.619 |
|  | SVC | 0.737 | 0.725 | 0.844 | 0.589 | 0.707 | 0.614 | 0.804 | 0.405 |
| MRMR | DT | 0.958 | 0.912 | 1.000 | 0.811 | 0.718 | 0.693 | 0.826 | 0.548 |
|  | LR | 0.731 | 0.696 | 0.862 | 0.505 | 0.689 | 0.636 | 0.870 | 0.381 |
|  | RF | 0.817 | 0.799 | 0.963 | 0.611 | 0.695 | 0.625 | 0.848 | 0.381 |
|  | SVC | 0.713 | 0.681 | 0.807 | 0.537 | 0.692 | 0.602 | 0.783 | 0.405 |
| Relief | DT | 0.879 | 0.814 | 1.000 | 0.600 | 0.713 | 0.670 | 0.913 | 0.405 |
|  | LR | 0.725 | 0.696 | 0.761 | 0.621 | 0.706 | 0.659 | 0.826 | 0.476 |
|  | RF | 0.736 | 0.701 | 0.853 | 0.526 | 0.723 | 0.614 | 0.848 | 0.357 |
|  | SVC | 0.714 | 0.681 | 0.734 | 0.621 | 0.716 | 0.659 | 0.783 | 0.524 |
| RFS | DT | 0.760 | 0.770 | 0.982 | 0.526 | 0.734 | 0.659 | 1.000 | 0.286 |
|  | LR | 0.736 | 0.686 | 0.771 | 0.589 | 0.683 | 0.591 | 0.804 | 0.357 |
|  | RF | 0.822 | 0.784 | 0.945 | 0.600 | 0.692 | 0.591 | 0.870 | 0.286 |
|  | SVC | 0.797 | 0.809 | 0.945 | 0.653 | 0.702 | 0.625 | 0.783 | 0.452 |
| SPEC | DT | 0.876 | 0.828 | 1.000 | 0.632 | 0.720 | 0.705 | 0.891 | 0.500 |
|  | LR | 0.714 | 0.681 | 0.697 | 0.663 | 0.668 | 0.636 | 0.717 | 0.548 |
|  | RF | 0.831 | 0.784 | 0.908 | 0.642 | 0.693 | 0.636 | 0.848 | 0.405 |
|  | SVC | 0.738 | 0.701 | 0.743 | 0.653 | 0.684 | 0.591 | 0.674 | 0.500 |
| Trace ratio | DT | 0.780 | 0.721 | 1.000 | 0.400 | 0.649 | 0.625 | 1.000 | 0.214 |
|  | LR | 0.739 | 0.701 | 0.817 | 0.568 | 0.704 | 0.568 | 0.783 | 0.333 |
|  | RF | 0.876 | 0.814 | 0.927 | 0.684 | 0.752 | 0.670 | 0.870 | 0.452 |
|  | SVC | 0.683 | 0.667 | 0.826 | 0.484 | 0.713 | 0.648 | 0.826 | 0.452 |
| **T2_edema** | | | | | | | | | |
| **Feature selection** | **Classifier** | **Training cohort**  **(n=204)** | | | | **Validation cohort**  **(n=88)** | | | |
|  |  | **AUC** | **ACC** | **SEN** | **SPE** | **AUC** | **ACC** | **SEN** | **SPE** |
| CFS | DT | 0.857 | 0.814 | 1.000 | 0.600 | 0.642 | 0.591 | 0.783 | 0.381 |
|  | LR | 0.697 | 0.667 | 0.697 | 0.632 | 0.657 | 0.614 | 0.717 | 0.500 |
|  | RF | 0.725 | 0.681 | 0.661 | 0.705 | 0.649 | 0.602 | 0.696 | 0.500 |
|  | SVC | 0.652 | 0.652 | 0.853 | 0.421 | 0.718 | 0.648 | 0.913 | 0.357 |
| CIFE | DT | 0.837 | 0.784 | 0.844 | 0.716 | 0.744 | 0.705 | 0.761 | 0.643 |
|  | LR | 0.776 | 0.735 | 0.798 | 0.663 | 0.701 | 0.648 | 0.804 | 0.476 |
|  | RF | 0.836 | 0.799 | 0.835 | 0.758 | 0.681 | 0.625 | 0.739 | 0.500 |
|  | SVC | 0.765 | 0.760 | 0.899 | 0.600 | 0.728 | 0.659 | 0.870 | 0.429 |
| CMIM | DT | 0.882 | 0.824 | 0.917 | 0.716 | 0.730 | 0.693 | 0.783 | 0.595 |
|  | LR | 0.784 | 0.750 | 0.936 | 0.537 | 0.751 | 0.670 | 0.913 | 0.405 |
|  | RF | 0.929 | 0.882 | 0.945 | 0.811 | 0.701 | 0.648 | 0.717 | 0.571 |
|  | SVC | 0.774 | 0.755 | 0.954 | 0.526 | 0.754 | 0.670 | 0.913 | 0.405 |
| DISR | DT | 0.876 | 0.809 | 0.872 | 0.737 | 0.725 | 0.682 | 0.761 | 0.595 |
|  | LR | 0.769 | 0.735 | 0.835 | 0.621 | 0.733 | 0.682 | 0.848 | 0.500 |
|  | RF | 0.937 | 0.882 | 0.963 | 0.789 | 0.710 | 0.614 | 0.761 | 0.452 |
|  | SVC | 0.738 | 0.750 | 0.908 | 0.568 | 0.760 | 0.670 | 0.891 | 0.429 |
| Fisher | DT | 0.856 | 0.784 | 0.972 | 0.568 | 0.753 | 0.693 | 0.957 | 0.405 |
|  | LR | 0.755 | 0.716 | 0.853 | 0.558 | 0.693 | 0.670 | 0.891 | 0.429 |
|  | RF | 0.886 | 0.814 | 0.853 | 0.768 | 0.741 | 0.682 | 0.761 | 0.595 |
|  | SVC | 0.759 | 0.765 | 0.936 | 0.568 | 0.703 | 0.682 | 0.870 | 0.476 |
| Gini | DT | 0.796 | 0.750 | 0.982 | 0.484 | 0.729 | 0.648 | 0.957 | 0.310 |
|  | LR | 0.782 | 0.701 | 0.615 | 0.800 | 0.703 | 0.659 | 0.652 | 0.667 |
|  | RF | 0.914 | 0.858 | 0.872 | 0.842 | 0.707 | 0.614 | 0.630 | 0.595 |
|  | SVC | 0.756 | 0.711 | 0.789 | 0.621 | 0.716 | 0.614 | 0.804 | 0.405 |
| ICAP | DT | 0.882 | 0.824 | 0.917 | 0.716 | 0.730 | 0.693 | 0.783 | 0.595 |
|  | LR | 0.784 | 0.750 | 0.936 | 0.537 | 0.751 | 0.670 | 0.913 | 0.405 |
|  | RF | 0.929 | 0.882 | 0.945 | 0.811 | 0.701 | 0.648 | 0.717 | 0.571 |
|  | SVC | 0.774 | 0.755 | 0.954 | 0.526 | 0.755 | 0.670 | 0.913 | 0.405 |
| JMI | DT | 0.872 | 0.804 | 0.835 | 0.768 | 0.695 | 0.648 | 0.761 | 0.524 |
|  | LR | 0.777 | 0.730 | 0.807 | 0.642 | 0.708 | 0.659 | 0.804 | 0.500 |
|  | RF | 0.838 | 0.799 | 0.844 | 0.747 | 0.662 | 0.636 | 0.739 | 0.524 |
|  | SVC | 0.777 | 0.760 | 0.862 | 0.642 | 0.730 | 0.670 | 0.870 | 0.452 |
| MCFS | DT | 0.932 | 0.882 | 1.000 | 0.747 | 0.686 | 0.670 | 0.783 | 0.548 |
|  | LR | 0.754 | 0.696 | 0.670 | 0.726 | 0.686 | 0.625 | 0.652 | 0.595 |
|  | RF | 0.839 | 0.804 | 0.908 | 0.684 | 0.682 | 0.670 | 0.848 | 0.476 |
|  | SVC | 0.680 | 0.676 | 0.798 | 0.537 | 0.688 | 0.636 | 0.826 | 0.429 |
| MIFS | DT | 0.904 | 0.853 | 0.982 | 0.705 | 0.692 | 0.670 | 0.804 | 0.524 |
|  | LR | 0.782 | 0.730 | 0.771 | 0.684 | 0.702 | 0.659 | 0.783 | 0.524 |
|  | RF | 0.760 | 0.730 | 0.844 | 0.600 | 0.646 | 0.636 | 0.783 | 0.476 |
|  | SVC | 0.777 | 0.760 | 0.862 | 0.642 | 0.731 | 0.670 | 0.870 | 0.452 |
| MIM | DT | 0.878 | 0.824 | 0.945 | 0.684 | 0.711 | 0.682 | 0.848 | 0.500 |
|  | LR | 0.780 | 0.740 | 0.927 | 0.526 | 0.739 | 0.648 | 0.870 | 0.405 |
|  | RF | 0.872 | 0.804 | 0.844 | 0.758 | 0.704 | 0.659 | 0.717 | 0.595 |
|  | SVC | 0.713 | 0.711 | 0.798 | 0.611 | 0.768 | 0.705 | 0.870 | 0.524 |
| MRMR | DT | 0.939 | 0.868 | 0.991 | 0.726 | 0.714 | 0.693 | 0.826 | 0.548 |
|  | LR | 0.782 | 0.730 | 0.761 | 0.695 | 0.702 | 0.659 | 0.783 | 0.524 |
|  | RF | 0.887 | 0.838 | 0.945 | 0.716 | 0.673 | 0.602 | 0.783 | 0.405 |
|  | SVC | 0.777 | 0.760 | 0.862 | 0.642 | 0.731 | 0.670 | 0.870 | 0.452 |
| Relief | DT | 0.860 | 0.843 | 1.000 | 0.663 | 0.715 | 0.659 | 0.935 | 0.357 |
|  | LR | 0.724 | 0.696 | 0.853 | 0.516 | 0.681 | 0.614 | 0.870 | 0.333 |
|  | RF | 0.861 | 0.824 | 0.963 | 0.663 | 0.686 | 0.614 | 0.826 | 0.381 |
|  | SVC | 0.714 | 0.686 | 0.862 | 0.484 | 0.693 | 0.614 | 0.891 | 0.310 |
| RFS | DT | 0.787 | 0.750 | 0.936 | 0.537 | 0.711 | 0.682 | 0.957 | 0.381 |
|  | LR | 0.751 | 0.696 | 0.862 | 0.505 | 0.694 | 0.625 | 0.891 | 0.333 |
|  | RF | 0.909 | 0.848 | 0.890 | 0.800 | 0.696 | 0.614 | 0.804 | 0.405 |
|  | SVC | 0.760 | 0.725 | 0.835 | 0.600 | 0.728 | 0.705 | 0.870 | 0.524 |
| SPEC | DT | 0.784 | 0.760 | 0.862 | 0.642 | 0.656 | 0.580 | 0.783 | 0.357 |
|  | LR | 0.663 | 0.637 | 0.606 | 0.674 | 0.663 | 0.614 | 0.696 | 0.524 |
|  | RF | 0.848 | 0.819 | 0.936 | 0.684 | 0.662 | 0.591 | 0.739 | 0.429 |
|  | SVC | 0.642 | 0.652 | 0.844 | 0.432 | 0.690 | 0.659 | 0.957 | 0.333 |
| Trace ratio | DT | 0.917 | 0.868 | 1.000 | 0.716 | 0.726 | 0.705 | 0.913 | 0.476 |
|  | LR | 0.803 | 0.735 | 0.679 | 0.800 | 0.687 | 0.625 | 0.696 | 0.548 |
|  | RF | 0.831 | 0.765 | 0.927 | 0.579 | 0.685 | 0.614 | 0.826 | 0.381 |
|  | SVC | 0.936 | 0.922 | 0.991 | 0.842 | 0.703 | 0.682 | 0.804 | 0.548 |

Note: AUC: area under curve; ACC: accuracy; SEN: sensitivity; SPE: specificity. The optimal strategy choosing was based on AUC meanwhile avoiding overfitting. Considering tumor margin was clearer than edema margin, the optimal strategy choosing was referred to tumor signature.

**Supplementary Table 5. Robustness analysis of HMDT in training and validation cohorts**

| **Model** | **Training cohort**  **(n=204)** | | | | **Validation cohort**  **(n=88)** | | | |
| --- | --- | --- | --- | --- | --- | --- | --- | --- |
|  | **AUC**  **(95% CI)** | **ACC** | **SEN** | **SPE** | **AUC**  **(95% CI)** | **ACC** | **SEN** | **SPE** |
| **Clinical**  **model** |  |  |  |  |  |  |  |  |
| **Grouping 1** | 0.813  (0.753,0.873) | 0.760 | 0.898 | 0.604 | 0.828  (0.744,0.912) | 0.704 | 0.851 | 0.537 |
| **Grouping 2** | 0.828  (0.771,0.885) | 0.745 | 0.725 | 0.768 | 0.782  (0.686,0.878) | 0.693 | 0.696 | 0.690 |
| **Grouping 3** | 0.822  (0.765,0.879) | 0.745 | 0.709 | 0.782 | 0.795  (0.699,0.891) | 0.727 | 0.711 | 0.750 |
| **Fusion signature** |  |  |  |  |  |  |  |  |
| **Grouping 1** | 0.976  (0.958,0.995) | 0.941 | 0.954 | 0.927 | 0.922  (0.863,0.980) | 0.864 | 0.936 | 0.780 |
| **Grouping 2** | 0.975  (0.959,0.992) | 0.931 | 0.963 | 0.895 | 0.923  (0.861,0.985) | 0.886 | 0.913 | 0.857 |
| **Grouping 3** | 0.971  (0.949,0.993) | 0.936 | 0.961 | 0.911 | 0.933  (0.881,0.985) | 0.875 | 0.923 | 0.806 |
| **HMDT** |  |  |  |  |  |  |  |  |
| **Grouping 1** | 0.981  (0.964,0.997) | 0.946 | 0.944 | 0.948 | 0.945  (0.896,0.994) | 0.875 | 0.936 | 0.805 |
| **Grouping 2** | 0.980  (0.964,0.996) | 0.941 | 0.936 | 0.947 | 0.936  (0.881,0.990) | 0.875 | 0.870 | 0.881 |
| **Grouping 3** | 0.978  (0.958,0.997) | 0.936 | 0.932 | 0.941 | 0.944  (0.897,0.992) | 0.898 | 0.904 | 0.889 |

**Note:** AUC: area under curve; CI: confidence interval; ACC: accuracy; SEN: sensitivity; SPE: specificity.

**Supplementary Table 6. 3-fold cross-validation for developed models**

| **Model** | **Training cohort** | | | | **Validation cohort** | | | |
| --- | --- | --- | --- | --- | --- | --- | --- | --- |
|  | **AUC (95%CI)** | **ACC** | **SEN** | **SPE** | **AUC (95%CI)** | **ACC** | **SEN** | **SPE** |
| **Clinical model** |  |  |  |  |  |  |  |  |
| **Fold-1** | 0.811  (0.751-0.872) | 0.754 | 0.693 | 0.819 | 0.828  (0.745-0.910) | 0.711 | 0.704 | 0.721 |
| **Fold-2** | 0.824  (0.765-0.882) | 0.768 | 0.962 | 0.544 | 0.796  (0.707-885) | 0.694 | 0.922 | 0.447 |
| **Fold-3** | 0.811  (0.751-0.872) | 0.749 | 0.800 | 0.689 | 0.817  (0.733-0.902) | 0.722 | 0.720 | 0.723 |
| **Radiomics model** |  |  |  |  |  |  |  |  |
| **Fold-1** | 0.964  (0.941-0.987) | 0.918 | 0.959 | 0.878 | 0.948  (897-0.999) | 0.928 | 0.966 | 0.872 |
| **Fold-2** | 0.954  (0.925-0.984) | 0.928 | 0.952 | 0.900 | 0.969  (0.939-0.999) | 0.908 | 0.941 | 0.872 |
| **Fold-3** | 0.960  (0.932-0.988) | 0.923 | 0.945 | 0.895 | 0.956  (0.918-0.994) | 0.907 | 0.913 | 0.902 |
| **Combined model** |  |  |  |  |  |  |  |  |
| **Fold-1** | 0.970  (0.948-0.992) | 0.933 | 0.942 | 0.923 | 0.964  (0.927-1) | 0.928 | 0.922 | 0.935 |
| **Fold-2** | 0.966  (0.941-0.992) | 0.943 | 0.952 | 0.933 | 0.969  (0.941-0.998) | 0.908 | 0.900 | 0.917 |
| **Fold-3** | 0.968  (0.945-0.990) | 0.923 | 0.941 | 0.904 | 0.966  (0.929-1) | 0.938 | 0.982 | 0.884 |

**Note:** AUC: area under curve; CI: confidence interval; ACC: accuracy; SEN: sensitivity; SPE: specificity.

**Supplementary Table 7. Diagnostic results from 8 neurosurgeons**

| **Typical case** | **Pathological result** | **Fusion radiomics signature result** | **HMDT predicted result** | **Fusion radiomics signature score** | **HMDT score** |
| --- | --- | --- | --- | --- | --- |
| **Case A** | 0 | 0 | 0 | 0.3075 | 0.0786 |
| **Case B** | 1 | 1 | 1 | 0.9313 | 0.9819 |
| **Case C** | 1 | 1 | 1 | 0.9344 | 0.9845 |
| **Case D** | 0 | 0 | 0 | 0.0000 | 0.001 |

**Note:** 0 represents for meningioma, and 1 represents for intracranial hemangiopericytoma. The fusion radiomics signature score and HMDT score both corresponds to the possibility of intracranial hemangiopericytoma.

**Supplementary Table 8. Diagnostic results from 8 neurosurgeons**

| **Neurosurgeon** | **Case A**  **Meningioma** | **Case B**  **IHPC** | **Case C**  **IHPC** | **Case D**  **Meningioma** |
| --- | --- | --- | --- | --- |
| **Junior surgeon 1** | 0 | 1 | 0 | 1 |
| **Junior surgeon 2** | 0 | 1 | 0 | 1 |
| **Junior surgeon 3** | 0 | 0 | 0 | 1 |
| **Junior surgeon 4** | 0 | 1 | 0 | 0 |
| **Junior surgeon 5** | 0 | 1 | 0 | 1 |
| **Senior surgeon1** | 1 | 1 | 0 | 1 |
| **Senior surgeon 2** | 0 | 1 | 0 | 0 |
| **Expert 1** | 0 | 1 | 0 | 1 |

**Note:** IIHPC: intracranial hemangiopericytoma. Case A and Case B were radiologically suspected as IHPCs. However, the pathological results showed A was meningioma and B was IHPC. Case C and Case D were radiologically suspected as meningiomas. However, the pathological results showed C was IHPC and D was meningioma. 1 represents the surgeon correctly diagnosed the case by radiological characteristics. 0 represents the surgeon wrongly diagnosed the case by radiological characteristics.

**Supplementary Material Reference**

1. Lambin, P., *Radiomics digital phantom.* CancerData. org, 2016.

2. F, T., et al., *Reproducibility of tumor uptake heterogeneity characterization through textural feature analysis in 18F-FDG PET.* Journal of Nuclear Medicine, 2012. 53(5): p. 693-700.

3. Sun, C. and W.G. Wee, *Neighboring gray level dependence matrix for texture classification.* Computer Vision Graphics & Image Processing, 1983. 23(3): p. 341-352.

4. Amadasun, M. and R. King, *Textural features corresponding to textural properties.* IEEE Transactions on systems, man, and Cybernetics, 1989. 19(5): p. 1264-1274.

5. Lambin, P., et al., *Radiomics: the bridge between medical imaging and personalized medicine.* Nature Reviews Clinical Oncology, 2017. 14(12): p. 749.

6. Guyon, I., et al., *Gene selection for cancer classification using support vector machines.* Machine learning, 2002. 46(1-3): p. 389-422.

7. Peng, H., F. Long, and C. Ding, *Feature Selection Based on Mutual Information: Criteria of Max-Dependency, Max-Relevance, and Min-Redundancy*. 2005: IEEE Computer Society. 1226.

8. Jakulin, A., *Machine learning based on attribute interactions*. 2005, Univerza v Ljubljani.

9. Torkkola, K., *Feature extraction by non-parametric mutual information maximization.* Journal of machine learning research, 2003. 3(Mar): p. 1415-1438.

10. Fleuret, F., *Fast binary feature selection with conditional mutual information.* Journal of Machine Learning Research, 2004. 5(Nov): p. 1531-1555.

11. Lin, D. and X. Tang. *Conditional infomax learning: an integrated framework for feature extraction and fusion*. in *European Conference on Computer Vision*. 2006. Springer.

12. Yang, H. and J. Moody. *Feature selection based on joint mutual information*. in *Proceedings of international ICSC symposium on advances in intelligent data analysis*. 1999. Citeseer.

13. Meyer, P.E. and G. Bontempi. *On the use of variable complementarity for feature selection in cancer classification*. in *Workshops on Applications of Evolutionary Computation*. 2006. Springer.

14. Battiti, R., *Using mutual information for selecting features in supervised neural net learning.* IEEE Transactions on neural networks, 1994. 5(4): p. 537-550.

15. Zhao, M., Z. Zhang, and T.W. Chow, *Trace ratio criterion based generalized discriminative learning for semi-supervised dimensionality reduction.* Pattern Recognition, 2012. 45(4): p. 1482-1499.

16. PEHRO, D. and D. Stork, *Pattern classification.* D Wiley-Interscience Publication, 2001.

17. Zhao, Z. and H. Liu. *Spectral feature selection for supervised and unsupervised learning*. in *Proceedings of the 24th international conference on Machine learning*. 2007. ACM.

18. Gilad-Bachrach, R., A. Navot, and N. Tishby. *Margin based feature selection-theory and algorithms*. in *Proceedings of the twenty-first international conference on Machine learning*. 2004. ACM.

19. Gini, C., *Variabilità e mutabilità.* Reprinted in Memorie di metodologica statistica (Ed. Pizetti E, Salvemini, T). Rome: Libreria Eredi Virgilio Veschi, 1912.

20. Nie, F., et al. *Efficient and robust feature selection via joint ℓ2, 1-norms minimization*. in *Advances in neural information processing systems*. 2010.

21. Cai, D., C. Zhang, and X. He. *Unsupervised feature selection for multi-cluster data*. in *Proceedings of the 16th ACM SIGKDD international conference on Knowledge discovery and data mining*. 2010. ACM.

22. Harrell, F.E., *Ordinal logistic regression*, in *Regression modeling strategies*. 2001, Springer. p. 331-343.

23. Liaw, A. and M. Wiener, *Classification and regression by randomForest.* R news, 2002. 2(3): p. 18-22.

24. Hearst, M.A., et al., *Support vector machines.* IEEE Intelligent Systems and their applications, 1998. 13(4): p. 18-28.
